# ABHD11, a new diacylglycerol lipase involved in weight gain regulation

**DOI:** 10.1371/journal.pone.0234780

**Published:** 2020-06-24

**Authors:** Johanna Escoubet, Mireille Kenigsberg, Murielle Derock, Veeranagouda Yaligara, Marie-Dominique Bock, Sandrine Roche, Florence Massey, Hélène de Foucauld, Charles Bettembourg, Anne Olivier, Antoine Berthemy, Joël Capdevielle, Richard Legoux, Eric Perret, Armelle Buzy, Pascale Chardenot, Valérie Destelle, Aurélie Leroy, Christophe Cahours, Sandrine Teixeira, Patrick Juvet, Pascal Gauthier, Michaël Leguet, Laurence Rocheteau-Beaujouan, Marie-Agnès Chatoux, Willy Deshayes, Margerie Clement, Mostafa Kabiri, Cécile Orsini, Vincent Mikol, Michel Didier, Jean-Claude Guillemot

**Affiliations:** 1 Sanofi Research and Development, Chilly-Mazarin, France; 2 Evotec, Toulouse, France; 3 Sanofi Research and Development, Toulouse, France; 4 Sanofi Research and Development, Montpellier, France; 5 Sanofi Research and Development, Industriepark Höchst, Frankfurt, Germany; "INSERM", FRANCE

## Abstract

Obesity epidemic continues to spread and obesity rates are increasing in the world. In addition to public health effort to reduce obesity, there is a need to better understand the underlying biology to enable more effective treatment and the discovery of new pharmacological agents. Abhydrolase domain-containing protein 11 (ABHD11) is a serine hydrolase enzyme, localized in mitochondria, that can synthesize the endocannabinoid 2-arachidonoyl glycerol (2AG) in vitro. In vivo preclinical studies demonstrated that knock-out ABHD11 mice have a similar 2AG level as WT mice and exhibit a lean metabolic phenotype. Such mice resist to weight gain in Diet Induced Obesity studies (DIO) and display normal biochemical plasma parameters. Metabolic and transcriptomic analyses on serum and tissues of ABHD11 KO mice from DIO studies show a modulation in bile salts associated with reduced fat intestinal absorption. These data suggest that modulating ABHD11 signaling pathway could be of therapeutic value for the treatment of metabolic disorders.

## Introduction

The prevalence of obesity is increasing and affects 650 million people, becoming one of the foremost global health threats [1] [2]. Obesity can seriously impair health through a broad range of complications such as cardiovascular diseases, type 1 and 2 diabetes, cancer, musculoskeletal disorders, psychosocial imbalances and reduced quality of life. Lifestyle modification is an integral part of the weight management journey, but is often insufficient on its own, and needs to be complimented by pharmacological and surgical add-on treatments to achieve greater and more sustainable weight loss, as appropriate. There are so far a limited number of pharmacological agents that are effective and safe for managing obesity [3]. As a result, there is a need to identify new targets and molecular therapeutics for the treatment of this metabolic disorder which is one of the most difficult public health issues our society has faced in recent years.

Targeting the endocannabinoid system by blocking cannabinoid receptors or enzymes responsible for their synthesis was considered a promising strategy to treat obesity and associated diseases. One of the physiological roles of the endocannabinoid system is the regulation of the metabolic homeostasis via central, but also peripheral, cannabinoid receptor 1 (CB1). Endocannabinoids play a role in the regulation of food intake by affecting feeding behavior and by acting directly on peripheral organs such as the liver, white adipose tissue and the gastrointestinal tract where CB1 is expressed. [4–7]. However, CB1 antagonists have shown limited benefit risk ratio in humans [8].

The endogenous ligand of CB1 and CB2, 2-Arachidonoyl glycerol (2-AG) [9, 10], is a full agonist and most abundant in the rodent brain [11]. 2-AG is a neuromodulator playing roles in learning and memory, anxiety, pain, appetite and weight gain [12].

2-AG levels are tightly controlled by different biosynthetic and degradative pathways. Several enzymes have been implicated in its catabolic pathway, Monoacylglycerol lipase (MAGL) being the principal hydrolase in rodent brain [13], but it can be also degraded by FAAH, ABHD6 or ABHD12 to arachidonic acid and glycerol [14].

In 1989, Farooqui et al. described 2-diacylglycerol lipases (DAGL) activities in bovine brain [15], one localized in plasma membrane and the other localized in microsomes. Both activities were inhibited by the lipase inhibitor RHC80267. The latter authors purified, but did not identify, a 27 kDa protein from the microsomal fraction and a 52 kDa protein from the plasma membrane fraction.

In 2003, Bisogno et al. identified by a bioinformatic approach and cloned two human DAG lipases: DAGLα and β of respectively 70 and 120 kDa [16]. Both proteins are expressed at the plasma membrane. They have four transmembrane domains and contain the serine hydrolase catalytic triad Ser, Asp, His with the serine included in the serine lipase motif GXSXG. These DAGLs generate 2-AG from DAG in a Ca2+ dependent manner. The main inhibitors described were non-specific lipases inhibitors, RCH80267 [17] and THL (tetrahydrolipstatin) [18]. Inhibitors of DAG lipase α were shown to reduce food intake and body weight in mice [19] but no compounds have demonstrated clinical efficacy so far.

In 2007, Rosenberger et al. further characterized substrate specificity and kinetic parameters of microsomal 29 kDa DAG lipase [20]. They showed that the enzyme hydrolyzes stearate in preference to palmitate from the sn-1 position of 1,2-diacyl-sn-glycerols and produced the CB-1 receptor ligand, 2-arachidonoyl-sn-glycerol.

In the present study, we have identified Abhydrolase domain-containing protein 11 (ABHD11) as a 29kDa DAG lipase that can synthesize *in vitro* the endocannabinoid, 2-Arachidonoyl glycerol (2-AG) from 1-stearoyl-2-arachidonoyl-sn-glycerol as a substrate. This mitochondrial enzyme is a new DAG lipase displaying a different tissue expression pattern and different cell localization from the previously characterized DAG lipases α and β.

There is no compelling monogenic genetic association between hABHD11 and human diseases. However, in the case of Williams-Beuren syndrome [21], about 25 genes are hemizygously deleted including ABHD11 gene. This rare developmental disease leads to supravalvular aortic stenosis, mental retardation, overfriendliness, and specific facial features. These patients also present metabolic abnormalities including decreased triglycerides (TG) [22] and lower body mass [23], but the direct association is not established yet.

To determine if this enzyme plays a role in obesity, mice deprived of ABHD11 (KO ABHD11) were phenotyped in a high fat diet (HFD) study and we have shown that the absence of ABHD11 leads to resistance to weight gain. KO ABHD11 mice under HFD display a lean phenotype and normal plasma parameters associated with blood bile salt level modification and reduced intestinal absorption of fat.

These *in vivo* data suggest that ABHD11 or ABHD11-associated signaling pathway could be an attractive target for weight gain regulation.

## Material and methods

### Liposome preparation and DAG lipase enzymatic reaction assay

ABHD11 enzymatic activity was measured by detecting the presence of the molecule referred to as m/z 287.1 (+) by mass spectrometry. This method is described, e.g. in Thomas et al. [24] and in Rimmerman et al. [25].

#### Liposome preparation

10 μL DAG (10 mg/ml in CH3CN) was mixed with 50 μL Lyso phosphatidyl choline (LPC-SIGMA at 5 mg/ml in CHCL3), evaporated to dryness, solubilized with 30 μL CHCI3 followed by 100 μL of homogenizing buffer (0.1 M MOPS pH 7.4, 0, 25% BSA) and then subjected to ultrasound using a warming ultrasonic bath (Deltasonic) in an open tube for 15 min at 37°C, allowing slow CHCL3 evaporation and liposome generation.

**DAGL activity.** was measured at 37°C for 20 hours with 90 μL of 100 mM MOPS pH 7.4, 100 μL of purified fraction from brain, or equivalent quantity of recombinant ABHD11, and 10 μL DAG in LPC liposome as substrate (150 μΜ final DAG) or variable amount for enzymatic characterization.

For PMSF and RHC80267 inhibition, preincubation was carried out for 30 min at 3 mM and 1 mM, respectively, before adding DAG. RHC80267 (Sigma Aldrich) was also used. The reactions were stopped by adding chloroform supplemented with internal standard 2-AG-D8 (Cayman chemicals) for extraction. After centrifugation (5 min at 1500g), the organic layers were collected and dried under vacuum. The residues were suspended with 50 μL of H20/CH3CN mix (80:20) and analyzed by high performance liquid chromatography/mass spectrometry in "MRM-like" mode.

#### LC-MSMS "MRM-like" experiment

The HPLC system consisted of an Ultimate (DIONEX) mounted with a NanoColumn POROS R1–150 mm x 75 μm, equilibrated with 70% eluent A (H20 0.2% HCOOH) and 30% eluent B (CH3CN 0.2% HCOOH). 2-AG was eluted with a linear gradient from 30% eluent B to 100% eluent B in 12 min with a flow rate of 200 nL/min, followed by an isocratic wash with 100% eluent B. Experiments were performed on a Q-TOF instrument (Waters-Micromass) in positive ionization mode operating in the MS/MS mode. MS/MS analyses were performed over a mass range of m/z 250–300 and mass set 379.29 (+) during the LC-MS run, at the retention time of 2-AG, in a window of 10 min. Energy collision was set to 28ev. The transition 379.29(+) -> 287.1 (+) was extracted for quantification purposes and normalized with m/z 637.3 (from LPC).

### Immunofluorescence cell staining

MCF7 cells were rinsed in PBS, fixed 10 min with 3.7% paraformaldehyde and permeabilized in 0.5% Triton in PBS for 15 min. After two washes in PBS, cells were incubated in Antibody Diluent with Background Reducing Components (DAKO) for 1 hour. Cells were then co-stained overnight at 4°C with anti-ABHD11 mouse antibody (Abnova, H00083451-B01P) diluted at 1/25 and anti-COX IV rabbit antibody (Abcam, ab16056) diluted at 1/500 in antibody diluent. After 2 washes in PBS, anti-mouse Alexa-488 and anti-rabbit Alexa-555 at 1/500 in antibody diluent were incubated for 1h. Finally, after 2 washes in PBS, nuclei were stained with DAPI followed by 2 further washes in PBS. Images were obtained with confocal microscope Leica DMI 3000.

### Mitochondria purification from mice brain

Mitochondria were isolated by Percoll density gradient using the protocol described by Sims et al. [26] and Western blot analyses were performed for VDAC-1 (mitochondrial marker), LDH, Calregulin and ABHD11.

### Generation of KO ABHD11 mice

To delete the entire ABHD11 gene sequence, a vector containing the LacZ-loxP-Ub1-em7-Neo-loxP cassette, flanked by endogenous mouse homology arms at 5' and 3', was constructed and introduced into C57BL6F1 ES cells. Successful transfection of ES cells replaced the entire coding region from ATG in exon1 to TAA in exon6 of the murine gene with LacZ-loxP-Ub1-em7-Neo-loxP cassette. LacZ was fused at the endogenous ATG. The entire deletion Size was 2,309 nt. Regeneron’s GEMM platform (VelociMouse®) was used to target ES cells and microinject them into mouse embryos. In brief, C57BL6F1 ES cells were electroporated with the linearized vector construct and positive clones were microinjected into 8-cell stage mouse C57BL6 embryos. Microinjected embryos were transferred to uteri of pseudo pregnant recipient females, weaned pups were scored, and high percentage chimera males were selected for mating with flp-positive C57BL6 females to generate F1 animals for further breeding. Wild type and KO animals were identified by genomic tail DNA probed with PCR applying the following primer pairs: WT-Fwd: 5’- GCGACAAACCCCAAGACTTC-3’; WT-Rev: 5’- AGTCCC CTGGCTGCT CTTC-3’; KO-Fwd: 5’-TTCTCAGTATTGTTTTGCCAAGTTCT-3’. Mice were kept and bred under specific pathogen-free conditions in the animal care facilities accredited by the Association for Assessment and Accreditation of Laboratory Animal Care International (AAALAC) and all mouse experiments were conducted in accordance with European guidelines for care and use of laboratory animals and were approved by the local ethics committee (Comités d'Ethique pour la Protection de l'Animal de Laboratoire C2EA‐24 and CEEA-22). Mice were housed (maximum 6 for females and 4 for males) in standard cages of 370cm^2^, containing bedding made of wood shavings. They were identifiable by a microchip system. The animal facility was under controlled conditions of light, temperature, pressure and humidity.

### DIO design

Three week-old mice were genotyped and fed with a standard diet (STD): 6% soybean oil corresponding to 14.8 kJ% fat (Ssniff, Germany). They were randomized at Week 6 on weight parameter to generate HFD and STD groups for each genotype. At this step, mice in the HFD group were switched from the STD to a HFD (47.4 kJ% fat) based on beef tallow, hydrogenated coconut oil and corn oil (Ssniff, Germany). Mice in the STD group were maintained on STD until the end of the study. Body mass and food intake were recorded weekly. To collect feces, mice were placed in individual metabolic cages for 24h with free access to food and drink. The feces were then weighed and frozen at -80°C until lipid extraction.

Three independent Diet Induced Obesity experiments, named DIO1, DIO2 and DIO3 have been performed on WT and KO ABHD11 female mice during this study. These three DIO were performed in the same experimental conditions, only the number of mice per group, the duration of the study and some read out were different between them.

#### DIO 1

This DIO involved 26 female and 24 male mice distributed in the following groups. Females: 7 KO mice under HFD, 6 KO mice under STD, 7 WT mice under HFD and 6 WT mice under STD. Males: 8 KO mice under HFD, 8 WT mice under HFD and 8 WT mice under STD (severe reduction of male KO mice birth led us to form only one group of KO mice, the HFD). Mice weight and food consumption were recorded from 4th to 27th week of age. Fasting insulin level and fasting glycemia were measured at Weeks 8, 12, 16, 20 and 24. Non-fasting insulin was recorded at 6, 10, 14, 18, 22 and 27 weeks of age. Oral glucose tolerance test was carried out at Week 15. Tissues were collected at the end of the study and flash frozen for endocannabinoid measurement by mass spectrometry.

#### DIO 2

This DIO involved 32 female mice distributed in the following groups: 8 KO mice under HFD, 8 KO mice under STD, 8 WT mice under HFD and 8 WT mice under STD. Mice weight was recorded from 6th to 31th week of age. Blood were collected for serum preparation at 6th and 30th weeks of age and used for metabolomics studies, in which 749 metabolites were simultaneously quantified. Tissues were collected for transcriptomic analysis at the end of the study (30 weeks of age).

#### DI0 3

This DIO involved 108 female mice distributed in the following groups: 27 KO mice under HFD, 27 KO mice under STD, 27 WT mice under HFD and 27 WT mice under STD. Mice weight and food consumption were recorded from 6th to 19th week of age. Feces collection was carried out at the 19th week of age.

At the end of the studies, mice were euthanized by pentobarbital administration. Tissues were flash frozen for transcriptomic analysis or 2-AG quantification.

For practical reasons and following the observation of similar phenotypes between male and female and a more thorough characterization of female group during DIO1, DIO 2 and DIO 3 were only performed on female. Most of data presented in this paper have been obtained from female mice. The weight curve of male mice from the DIO 1 is presented in Supporting Information.

### Insulin recording

Insulin was measured in plasma with Mouse Ultrasensitive Insulin ELISA (Alpco) according to the manufacturer’s instruction.

### Blood glycemia recording

Glucose levels were measured with a blood glucose meter BG Star® (Sanofi)

### Oral glucose tolerance test

The test was performed with overnight fasting mice during DIO1. A solution of 2g glucose/kg was orally administrated to mice at 10mL/kg. Blood was collected from tail just before the administration of glucose and after 30 min, 1h, 1h30 and 2h. Glycemia was measured with BG Star (sanofi).

### Insulin sensitivity test

The test was performed with 2 hours-fasting mice during DIO1 at Week 26. The concentration of insulin (one Unit/kg) was chosen to induce a significant reduction of blood glucose level without risking of animal loss. Insulin (Umuline Rapide®, Lilly) was intraperitoneally administered to mice. Glycemia was measured just before the administration of insulin and after 15, 30, 45 min and 1h30.

### HOMA-IR index

The HOMA-IR (homeostasis model assessment of insulin resistance) index was calculated as [fasting glucose in mg/dL] *[fasting insulin in mU/L]/405 to assess insulin resistance.

### Statistical methodology for DIO generated data

A three-way analysis of variance was performed with Genotype, Diet as factors and Time as repeated factor. Then, when appropriate, post-hoc analyses were performed to see the effect of the diet and of the genotype on the studied parameter. For parameters Body weight, Glycemia, delta-insulin sensitivity versus baseline, a three-way analysis of variance was performed on data. For parameters delta-OGTT versus baseline and Insulin, a three-way analysis of variance was performed on rank-transformed data. Significance level was set to 5% (pvalue<0.05).

### RNA extraction and quality assessment for mice liver RNA transcriptomic analysis

Frozen mice liver samples from DIO2 were thawed on ice and used for total RNA extraction: 700 μL of QIAzol Lysis Reagent (Qiagen) was added to 20 to 30 mg of liver tissue in tissue homogenizing tubes (Precellys) and homogenized using a Precellys 24 lyser. Then 140 μL chloroform was added, mixed for 15 sec and centrifuged 15 min at 12000 g at 4°C. The upper aqueous phase was discarded and RNA fraction was isolated using QIAcube HT-Kit and QIAExtractor (Qiagen). RNA quality was determined using RNA 6000 Nano kit and 2100 Bioanalyzer (Agilent). All RNA samples had an RNA integrity number (RIN) between 7 and 9.

### Microarray and data analysis

About 100 ng total RNA was processed for Microarray as described by manufacturer using SurePrint G3 Mouse Gene Expression v2 8x60K Microarray chips (Agilent). Microarray files were analyzed using Array studio (OmicSoft Corp. Qiagen). Data analysis includes 1) generation of raw signal intensities from g-Processed signal, 2) setting threshold/ adding constants (raw signal intensities < 5 were set as 1), 3) combining multiple probes based on Mapping gene name, 4) Log2 transformation and 5) 75^th^ Quantile normalization. Non-parametric tests were performed on normalized data and only samples which passed QC were used for two-way ANOVA.

### Metabolomic study on mouse serum

Mice sera were flash frozen and delivered to Metabolon, Inc (617 Davis Drive, Suite 400, Durham, NC 27713) for metabolomics studies. In brief, samples were extracted and split into equal parts for analysis on the LC/MS/MS and Polar LC platforms. Proprietary software was used to match ions to an in-house library of standards for metabolite identification and for quantitation by peak area integration.

### Omics analysis of data

Both metabolomics and transcriptomics data were integrated with AskOmics (Garnier et al. 2017). This allowed us to detect significant changes in the metabolome of the KO mice, supported by transcriptomics significant changes in liver that could not have been detected by analyzing the datasets separately (metabolome only or transcriptome only)

We designed a data-driven model to load all the results of both studies in the same repository using AskOmics [27] (S6 Fig, in S1 Appendix).

To link genes to metabolites, all paths were computed between the Compound (metabolite) and Gene classes of the Kegg and Reactome databases, using the RDF version provided by Bo2RDF [28]. This integration work allowed us to query all data to identify genes and metabolites having a biological relation and also being affected by the genotype and/or the diet.

### Lipid extraction and dosage from feces

Feces from each mouse of DIO3 were resuspended in a normal saline solution and crushed with a spatula. Then, the same volume of chloroform/methanol solution (2:1) was added to proceed with the lipid extraction. The suspension was homogenized and then centrifuged 10 min at 1000g. To collect the lipid phase, the bottom of the tube was pierced and the lipid phase was collected in a pre-weighed glass vial. Solvent from the lipid phase was evaporated under a hood for 24h. Then, the vials containing the lipids which had been extracted from feces, were weighed again to obtain the lipid mass. They were further resuspended in cholesterol buffer 10X (600μL assay buffer/g of feces) (Cayman Chemical) by strong vortexing.

Total cholesterol concentration was quantified in lipid feces suspensions using a colorimetric assay (Cayman Chemical) according to the manufacturer’s instruction.

Triglycerides concentration was quantified in lipid feces suspensions using a colorimetric assay (Cayman Chemical) according to the manufacturer’s instruction.

Fatty acid concentration was quantified in lipid feces suspensions using a fluorimetric assay (Biovision) according to the manufacturer’s instruction.

## Results

### Purification, biochemical characterization, expression pattern and sequence analysis of ABHD11

#### ABHD11 is able to catalyze the hydrolysis of 1-stearoyl 2-arachidonoylglycerol

Starting from a whole pig brain homogenized in a detergent, we purified a 29 kDa protein following a protocol described by Farooqui et al. [15]. The isolation procedure is summarized in S1A Fig of S1 Appendix. We first used an activity-based protein profiling (ABPP) assay with a serine hydrolase probe (Fluorophosphonate, FP-bodipy) and then an enzymatic assay by 2-AG mass spectrometry (MS) titration to follow DAG lipase activity throughout the different purification steps.

S1B Fig in S1 Appendix of Supporting Information shows the detection of a 29 kDa band from pig brain, which fluoresces only in the absence of a serine hydrolase inhibitor PMSF. After extraction, soluble 29 kDa protein does not require any detergent and conventional purification steps can be carried out. We purified to homogeneity this protein with a DAG lipase activity. LC-MS analysis of the labelled band allowed the identification of ABHD11 tryptic peptides (S1E Fig of S1 Appendix).

The sequence encoding full length human ABHD11 was further cloned in a mammalian cell expression vector and the protein expressed in HEK293 mammalian cells. Triton X100 detergent extracts from HEK293 cells transfected with ABHD11 were purified according to a similar procedure (S1A Fig, in S1 Appendix). The Heparin-Sepharose elution fraction was submitted to ABPP. The transfected ABHD11 was detected only in the absence of PMSF (S1C Fig, in S1 Appendix), with a molecular weight corresponding to a mature form of the protein around 30 kDa. Recombinant ABHD11 protein was further purified to homogeneity and characterized by MS (S1D Fig, in S1 Appendix). The observed molecular mass of this recombinant protein agrees with the molecular mass of 30278 +/-3 Da corresponding to the polypeptide described in S1E Fig. The MS profile also illustrated the high degree of purity obtained.

Purified hABHD11 was further characterized following in-solution trypsin digestion by peptide fingerprinting and tandem LC MSMS. LC MSMS experiments analyses unambiguously identified a peptide having a VPAPSSSSGGRGGAEPR sequence corresponding to the N-terminal mature form of the protein, deleted from the 33 amino acid signal peptide (S1E Fig, in S1 Appendix).

**Fig 1:**
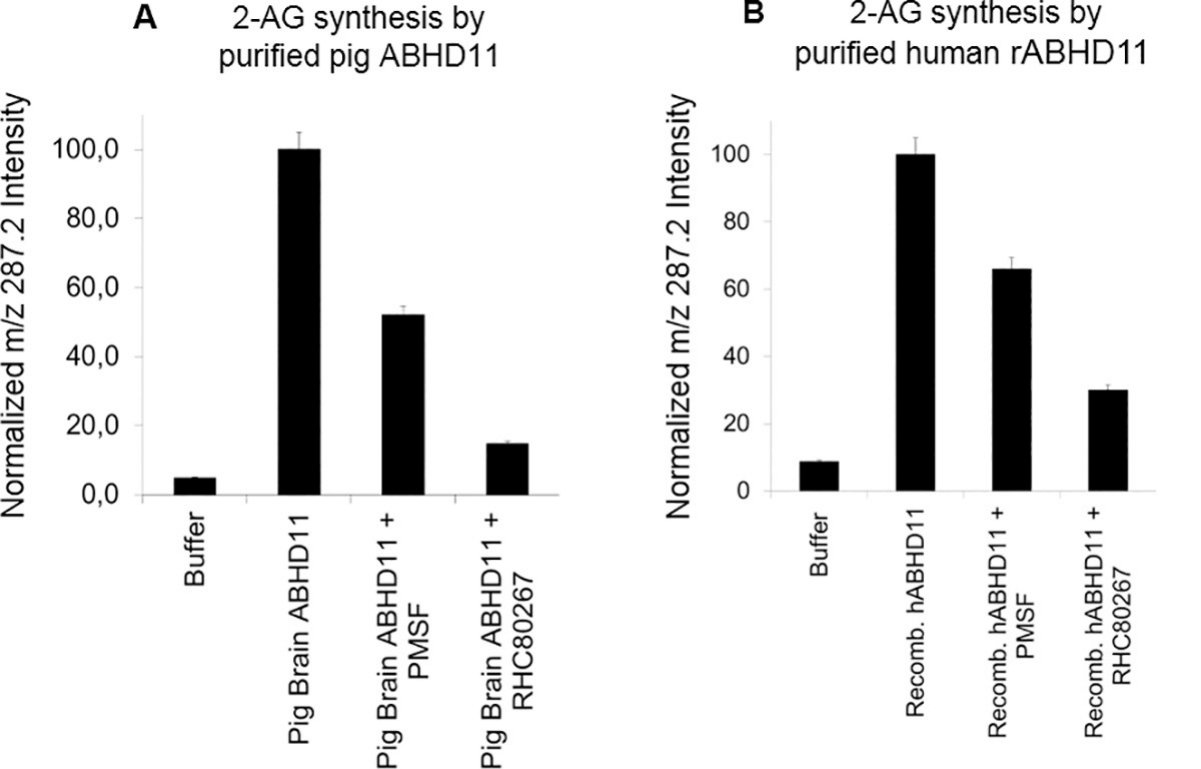
Purified ABHD11 is able to catalyze the hydrolysis of diacylglycerol. Purified ABHD11 from pig brain A- or human recombinant ABHD11 (rABHD11) B- were tested in DAG lipase assay: pre-incubation with inhibitors PMSF (3mM) or RHC80267 (1mM) or vehicle for 30 minutes, followed by 6hr DAG lipase incubation with DAG-encapsulated liposomes. Mass spectrometry analysis of 2-AG was then performed. All data are normalized on m/z 287.2 intensity. They represent the average of 2 individual experiments and are expressed as mean +/-SD.

DAG lipase assay was performed on purified recombinant human ABHD11, with or without preincubation with the serine hydrolase inhibitors PMSF or RHC80267. Following incubation of the protein with DAG-encapsulated liposome, targeted mass spectrometry analysis showed 2-AG synthesis was inhibited in presence of both serine hydrolase inhibitors (Fig 1A and 1B).

The kinetic parameters of recombinant human ABHD11 produced from HEK293 cells were determined using 1-stearoyl-2-arachidonoyl-sn-glycerol as a substrate. The calculated mean Vmax value was 2500 nmol/min/mg protein. The calculated mean Kmax value was 18.6μΜ (S1F Fig, in S1 Appendix). These results were in the same range as the bovine brain DAGL described by Rosenberger et al. [20], Vmax: 1900 or 616 nmol/min/mg and Km: 55 or 63μΜ, according respectively to the phosphorylated or non-phosphorylated forms of the enzyme, but these Vmax and Km values are much more potent than those of the two DAGL α and β published by Bisogno et al. [16] with Vmax of 33.3 +/- 4.5 and 3.4 +/- 0.2 nmol/min/mg protein and Km: 154.7+/- 19,1 and 74,1 +/- 4.9μΜ, respectively.

#### ABHD11 mitochondrial expression

**Fig 2:**
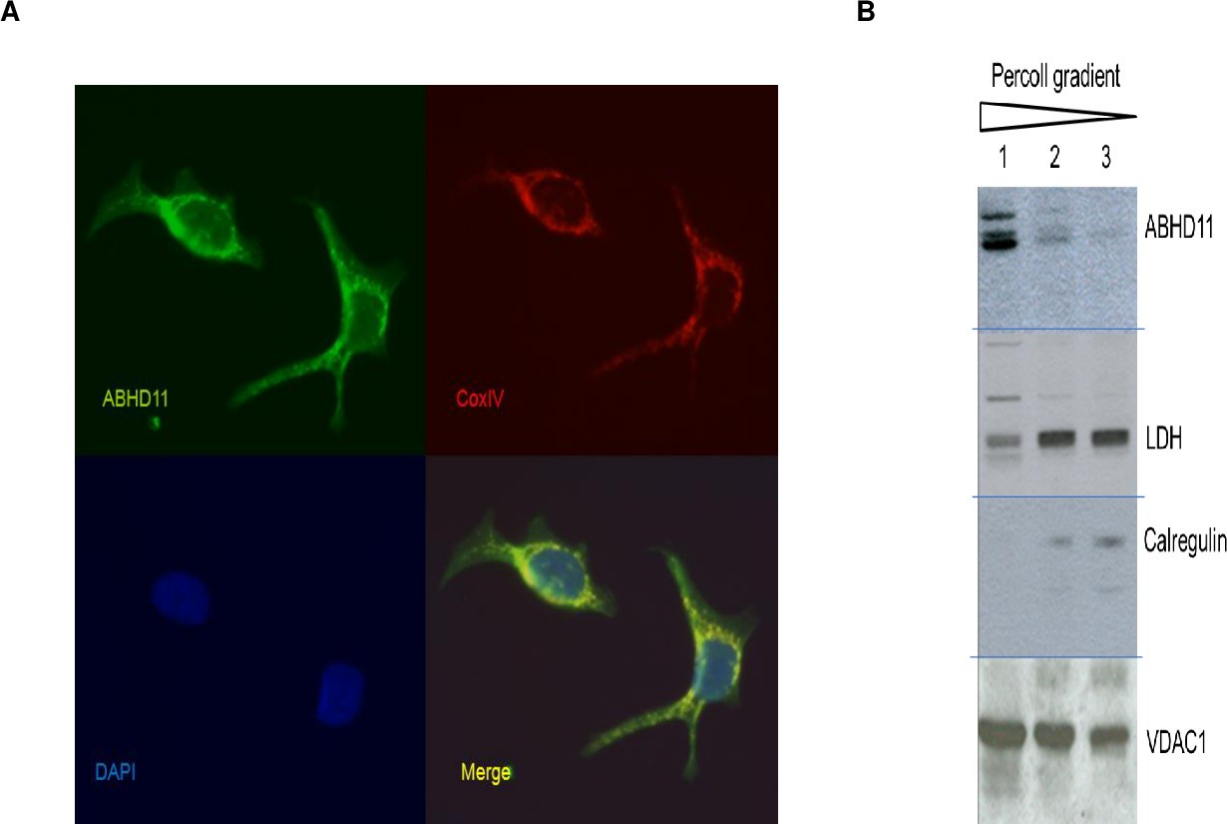
Mitochondrial expression of ABHD11 in vitro and in vivo. A- ABHD11 and COX4 co-localize in human breast adenocarcinoma MCF7 human cell line. Cells were fixed and stained with anti-ABHD11 and anti-COX4 antibodies following by incubation in DAPI (nuclei staining). Images were obtained by Leica DMI3000 confocal microscope. Representative images were captured at objective magnifications of ×40.B- mouse brain ABHD11 is in mitochondrial fraction. Purification of mitochondria from whole mouse brain by ultracentrifigation on gradient percoll density. Mitochondrial enrichment was tracked by analysis of the endoplasm reticulum protein (calregulin), cytoplasm protein L-lactate dehydrogenase A chain (LDH), and mitochondrial protein Voltage-dependent anion-selective channel protein 1 (VDAC-1). Lane 1 contains the highly enriched mitochondrial fraction. Lane 2 & 3 accumulated at the top of the gradient and contain the majority of synaptosomes, endoplasmic reticulum and cytosolic proteins. Blot images have been spliced (horizontal lines) and rearranged in preparing the figure.

The ABHD11 gene sequence contains a mitochondrial signal peptide at the N-terminus. Mitochondrial expression of ABHD11 was confirmed by immunofluorescence in different cancer cell lines, expressing endogenous ABHD11 protein as breast adenocarcinoma MCF7 (Fig 2A) and hepatocellular carcinoma HepG2 or colon adenocarcinoma SW480, in which the native ABHD11 immuno-signal co-localizes with COX4 (data not shown for these two last cell lines).

To further confirm mitochondrial expression in native tissues, mitochondria were isolated from mouse brain. Mitochondrial enrichment was tracked by analysis of endoplasmic reticulum, cytoplasmic and mitochondrial markers. As shown in Fig 2B, ABHD11 is contained within the highly enriched mitochondrial fraction, confirming previous localization studies.

#### Sequence analysis and comparison with other proteins

As previously described, ABHD11 presents a lipase motif, located from amino acid 94 to amino acid 98, and the catalytic triad S96, D192 and H251 (S1E Fig, in S1 Appendix). Surprisingly, the sequence of the ABHD11 DAG lipase is different from the sequences of DAGL-α (6.2% identity) and DAGL-β (9% identity), the sole diacylglycerol lipases known in the art to produce 2-AG *in vivo* (S2A Fig, in S1 Appendix).

Orthologs of ABHD11 exist in primates and mammals, but also in various microorganisms such as yeast (S. cerevisiae and K. lactis), filamentous fungi (N. crassa, A.gossypii, M. grisea, S.pombe), insect (D. melanogaster), parasites (P. falciparum, A. gambiae), domestic fowl (G. gallus), fish (D. rerio), nematodes (C. elegans) and amphibians (X. laevis) (S2B Fig, in S1 Appendix).

#### Expression profile of ABHD11 in human tissues

ABHD11 displays different distribution profiles from DAGL α and β mRNA expression and reveals a more ubiquitous distribution than the two other DAG lipases (S2A Fig, in S1 Appendix). Most investigated human tissues reveal noticeable levels of ABHD11 mRNAs with higher amounts detected in small intestine, prostate and thyroid, while aorta and colon tissues exhibit weak expression levels.

### Characterization of KO ABHD11 mice

Some inhibitors of ABHD11 serine hydrolase activity have been described [29–31] but their *in vitro* selectivity and *in vivo* activity remain unknown. To determine the role of ABHD11 beyond its enzymatic activity and evaluate its potential as a therapeutic target, we developed a somatic KO mouse model with deletion of the entire ABHD11 gene sequence. RT PCR and Western blots were performed to verify the absence of ABHD11 expression in KO mice (S14 Fig in S1 Appendix). The role of ABHD11 was first investigated *ex vivo* on isolated mitochondria and subsequently was investigated *in vivo* in a DIO study, performed in mice.

#### Ex vivo exploration of mitochondrial activity

The mitochondrial expression of ABHD11 led us to evaluate a functional role of this enzyme in mitochondria. Mitochondrial respiration profiles were compared in WT and KO ABHD11 mice in different *ex vivo* models including 1) isolated liver mitochondria, 2) mouse embryonic fibroblasts (MEFs), 3) primary hepatocytes, using both mitostress “normal” conditions and a beta oxidation assay that allowed both mitochondrial respiration and beta oxidation to be monitored (S3, S4, S5 Figs, in S1 Appendix).

Overall no difference in respiration profiles between WT and KO mouse cellular models were observed.

#### Mild differences between KO and WT ABHD11 mice

KO ABHD11 mice are viable and only a slight but significant reduction in body weight has been noticed in males exclusively. In addition, less abdominal fat deposit was observed in KO as compared to WT male animals (S6 Fig, in S1 Appendix).

Blood biochemical analyses were conducted in 9 week-old male mice and did not reveal KO genotype effects on any of the measured metabolic parameters, including LDL, HDL, total cholesterol, glucose, triglyceride, urea, creatinine, phosphor, calcium or bilirubin levels (S7A Fig, in S1 Appendix).

As both mitochondria and endocannabinoid systems are involved in regulating energy balance, ABHD11 KO mice were further evaluated in locomotor and muscular strength assays (S7B and S7C Fig, in S1 Appendix) with no significant difference observed, as compared to their control WT littermate animals. In addition, no change in rectal temperature was also noticed (37.5+/-0.2°C both for WT and KO male mice, n = 10).

A study was conducted on WT and KO mice (n = 6 per genotype) to determine any macroscopic and histological modification across tissues (skin, white and brown fats, genital organs, urinary tract, digestive tube, liver and gallbladder, pancreas, lymphoid organs, brain, eyes, optic nerves, endocrine system, cardio-respiratory system, skeletal muscle…). Only some eye defects were reported, including iris synechiae, melanophagy in the iris or the ciliary body, cataract (with reduced lens size when advanced) and retinal atrophy in KO mice. Therefore, 9-week old male KO mice were evaluated across sensory vision and touch escape tests (S7D and S7E Fig, in S1 Appendix). These investigations indicated that such ophthalmologic defects led to blindness. This vision defect observed in KO mice is probably linked to dysfunction occurring during embryo development. This defect has no influence on food intake as mice use their sense of smell for feeding. This has been controlled for measuring the food intake in the next DIO experiments.

### KO ABHD11 mice resist DIO and present normal biochemical parameters

#### KO ABHD11 mice resist weight gain induced by HFD

**Fig 3:**
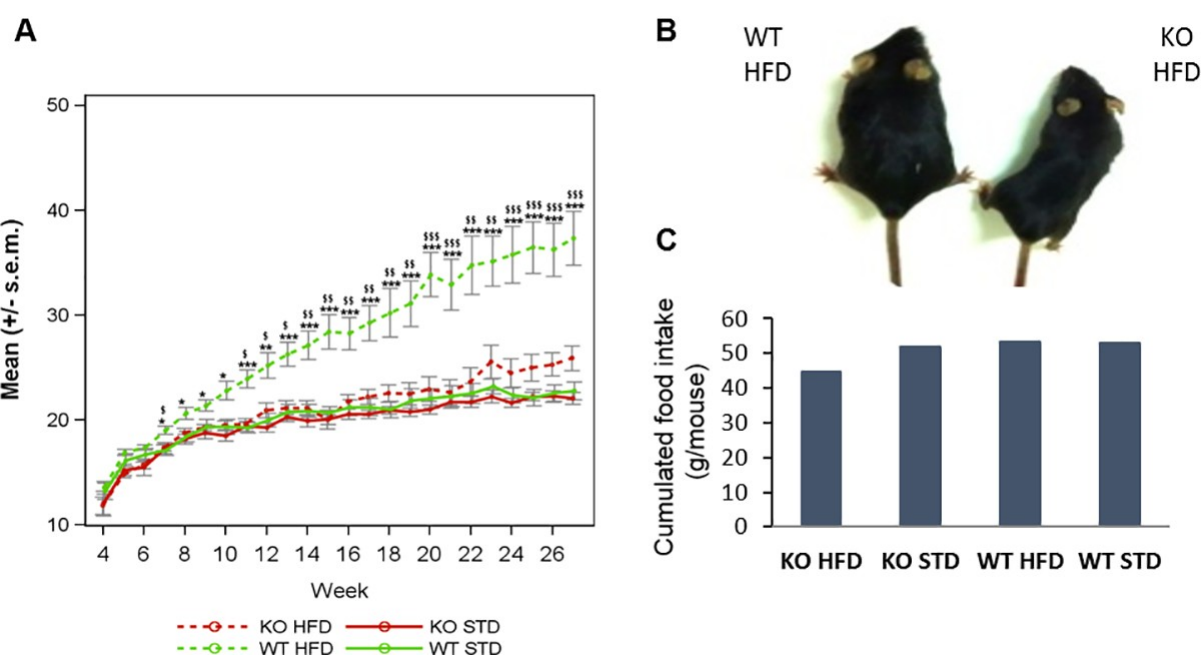
WT and KO ABHD11 female mice monitored in DIO1. WT and KO mice were separated in 4 groups according to each diet (STD or HFD) A- The weight of mice (grams) was recorded weekly. The curves represent means +/- s.e.m. at each day for each group (n = 6–7). *: pvalue<0.05, **: pvalue<0.01, ***: pvalue<0.001: obtained using a post-hoc analysis to compare HFD versus STD group in WT genotype after a three-way ANOVA with repeated measures on time factor ¤: pvalue<0.05, ¤¤: pvalue<0.01, ¤¤¤: pvalue<0.001: obtained using a post-hoc analysis to compare HFD versus STD group in KO genotype after a three-way ANOVA with repeated measures on time factor $: pvalue<0.05, $$: pv alue<0.01, $$$: pvalue<0.001: obtained using a post-hoc analysis to compare WT versus KO group in HFD group after a three-way ANOVA with repeated measures on time factor #: pvalue<0.05, ##: pvalue<0.01, ###: pvalue<0.001: obtained using a post-hoc analysis to compare WT versus KO group in STD group after a three-way ANOVA with repeated measures on time factor.B- Representative photo of a WT mouse and a KO ABHD11 mouse under HFD at the end of DIO. C- Cumulative food intake measured weekly during 24h from week 5 to week 27 (N = 1 cage per group).

As expected, WT mice under HFD presented a significantly higher weight compared to STD (standard diet) (p value <0.05 from Week 7) (Fig 3, study performed on female mice). In contrast, ABHD11 KO mice were resistant to diet-induced obesity, as no significant difference in mice weights were observed between the two regimens in DIO1. A strong resistance to weight gain, even not complete, is also observed in DIO2 and DIO3, showing the robustness of the genotype effect (S8A-S8B Fig, in S1 Appendix). Furthermore, DIO1 performed on both sexes, showed no sex difference in the resistance to DIO phenotype observed in KO mice. A significant lower weight was observed in KO HFD group compared to WT HFD group from Week 8 (S8D Fig, in S1 Appendix).

Due to the duration of the study, mice could not be kept in individual cages. Food and water consumption was evaluated by cage. As shown in Fig 3C, there were few differences in the cumulative food intake, whatever the genotype or the regimen. Only a slightly less food intake was measured in KO mice under HFD. This likely represents an artefact, as this observation was not reproduced in another DIO study with higher numbers of mice and cages (S8C Fig, in S1 Appendix). Globally, all mice ate the same quantity of food, showing that the body weight phenotype observed is not linked to food intake. The same observation was made for water consumption (data not shown).

#### Glycemia control

**Fig 4:**
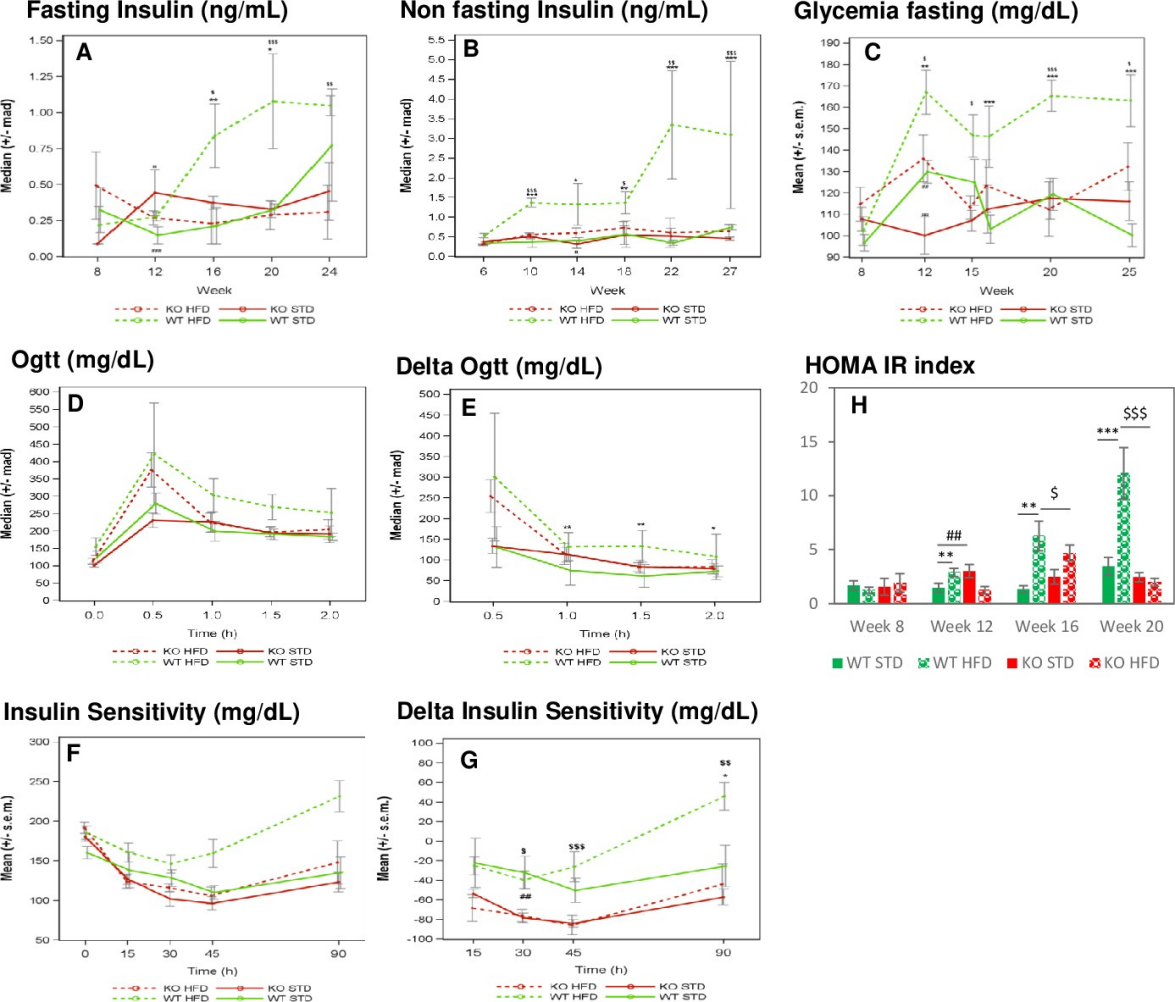
Measure of blood insulin and glycemia in mice of DIO1 in basal conditions (fasting or non fasting) and after glucose or insulin administration. n = 6–7 mice/group. A- Blood insulin evolution measured in non fasting mice, B- Blood insulin evolution measured in fasting mice, C- Blood glycemia evolution measured in fasting mice, D-E- Oral Glucose Tolerance test performed at 15 weeks of age: Glycemia measured after oral glucose tolerance administration (D), representation of delta glycemia level (compared to glycemia measured at T0) (E), F-G- Insulin sentitivity test: Glycemia measured after IP insulin administration (F), representation of delta glycemia level (compared to glycemia measured at T0) (G). H**-** HOMA-IR (homeostasis model assesment of insulin resistance) index. Fig A, B, C, D, E: *: pvalue <0.05, **:pvalue <0.01, ***:pvalue <0.001: obtained using a post-hoc analysis to compare HFD versus STD group in WT genotype after a three-way ANOVA with repeated measures on time factor **¤**: pvalue <0.05, **¤¤**:pvalue <0.01, **¤¤¤:**pvalue <0.001: obtained using a post-hoc analysis to compare HFD versus STD group in KO genotype after a three-way ANOVA with repeated measures on time factor $: pvalue <0.05, $$:pvalue <0.01, $$$:pvalue <0.001: obtained using a post-hoc analysis to compare WT versus KO group in HFD group after a three-way ANOVA with repeated measures on time factor #: pvalue <0.05, ##:pvalue <0.01, ###:pvalue <0.001: obtained using a post-hoc analysis to compare WT versus KO group in STD group after a three-way ANOVA with repeated measures on time factor. Fig H: pvalues obtained using a two-way ANOVA analysis.

*Fasting and non-fasting insulin level study*. Fasting and non-fasting blood insulin levels was monitored every four weeks from week 8 to week 24 for fasting conditions (Fig 4A) and from week 6 to week 27 for non-fasting conditions (Fig 4B).

In fasting conditions, a significantly higher insulin level was observed in WT mice under HFD, compared to STD, both at weeks 16 and 20. This difference was not significant at week 24, likely due to high heterogeneity present in the STD group at this time point.

In non-fasting conditions, significant higher circulating insulin levels were measured in WT animals under HFD, compared to STD, as early as from week 10 to the last measurement at week 27.

These increases of insulin level linked to the HFD, in fasting and non-fasting conditions, validate the DIO model. ABHD11 KO mice under HFD did not exhibit such DIO-dependent hyperinsulinemia. ABHD11 KO mice under HFD presented a significantly lower insulin level than WT from week 16 in fasting conditions and at Week 10 and from week 18 in non-fasting conditions.

*Fasting glycemia study*. Fasting glycemia was monitored every four weeks from week 8 to week 25 (Fig 4C). In WT mice, except at week 15, we observed a significantly higher glucose level under HFD compared to STD from week 12 and thereafter (p value <0.01 at week 12, and p value <0.001 at weeks 16, 20 and 25). In contrast, except for the week 12 time point, no difference in fasting glycemia levels was noticed in the ABHD11 KO group, whatever the regimen, indicating that mice with a deleted ABHD11 gene are resistant to DIO-induced hyperglycemia.

*Oral Glucose tolerance test (oGTT)*. To evaluate the capacity of KO ABHD11 mice to control their blood glycemia, we performed an oGTT at week 15 (Fig 4D). In all mice groups, a robust and transient increase in blood glycemia was observed with a peak at 30 minutes following oral glucose administration, then decreasing 30 to 90 min thereafter. As shown in Fig 4E, in WT mice, blood glucose levels were significantly higher in the HFD than in STD groups at 1, 1.5 and 2 hours post glucose administration, indicating that normalization of glycemia required longer time in the HFD than in STD groups. Interestingly, in the KO group, there was no significant difference observed between the regimens. These results further revealed a preserved glucose homeostasis in KO ABHD11 mice under HFD.

*Insulin sensitivity test*. As shown in Fig 4F, an acute administration of insulin led to a rapid decrease in the concentration of blood glucose. 30 minutes after injection, such blood glucose reduction was significantly higher in ABHD11 KO mice, compared to WT mice, whatever the diet, and was still elevated in the HFD group 45 and 90 minutes after the administration of insulin. Statistical analyses were performed on delta glycemia levels revealing no diet effect, whatever the genotype tested, except in the WT group 90 minutes after injection (p value <0.05). ABHD11 KO mice are more responsive to an acute insulin administration than WT mice. HOMA-IR index (homeostasis model assessment of insulin resistance) showed that under HFD, KO mice have lower values than WT from week 16.

Taken together, these observations revealed that ABHD11 KO mice are resistant to obesity induced by HFD and present normal blood parameters.

### No difference in 2-AG levels measured in mouse tissue

Since ABHD11 can catalyze *in vitro* the hydrolysis of DAG to 2-AG, we further compared endogenous 2-AG levels in tissues from KO and WT mice under standard diet conditions to understand the mechanism of action of ABHD11. Firstly, no difference in 2-AG content was observed in brain tissues such as cortex, hippocampus and olfactory bulbs (n = 3 animals/genotype). 2-AG levels were then further measured in the cerebral cortex, liver, heart, gastrocnemius muscle, pancreas and in fat tissues in KO/WT female mice at the end of DIO study (n = 6 or 7 animals/group). Similar to the standard diet, we globally could not report any significant change in 2-AG levels across the tissues investigated (S9 Fig, in S1 Appendix). In addition, no difference in 2-AG level was found in mitochondria isolated from mouse liver.

### Investigating the biological mechanism underlying weight gain resistance of KO AHBHD11 mice—Omics analysis

Additional unbiased transcriptomic and metabolomic studies were conducted to further investigate biological mechanisms underlying the weight gain resistance of the KO mice. Mice weight recording is reported in supplemental data (S8A Fig, in S1 Appendix).

#### Transcriptomic analysis of liver samples

**Fig 5:**
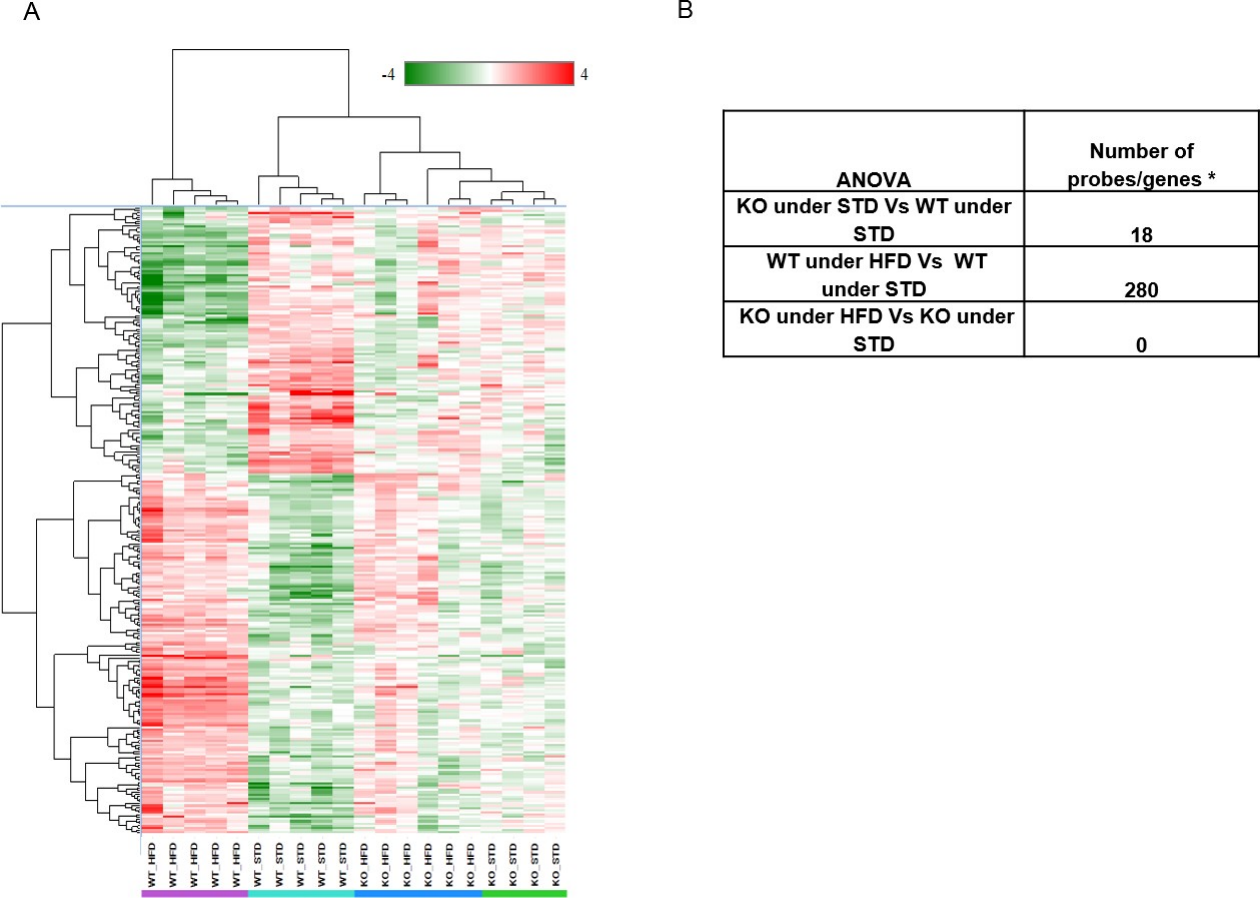
Transcriptomic analysis of mice liver from DIO 2. A- Hierarchical clustering on differentially expressed genes in liver samples from WTHFD vs WTSTD. Anova between WTHFD vs WTSTD yielded differential expression of 280 probes (genes/lincRNA having foldchange of ±2 with False Discovery Rate <0.05). B**-** number of genes/probes with Fold change of above +2 or below -2 with FDR 0.05.

Transcriptomic profiling was performed on liver samples from a DIO animal cohort (S9 Fig, in S1 Appendix). Genotype and diet effects on gene expression were analyzed by two way ANOVA (Fig 5 and S1 Table in S2 Appendix for details). The liver transcriptomic pattern of KO vs WT under STD was first compared. Only 18 transcripts were found to be differentially regulated while noting that genes adjacent to Abhd11 (Cldn3 and Stx1a) were dysregulated in KO under STD. The observed effect could be attributed to the polar effect of Abhd11 deletion, but not due to mutation effect itself. Apart from these genes, only CD36, a key enzyme involved in the uptake of fatty acids is upregulated 3 fold in KO under STD.

Then, the impact of diet on transcriptomic profile of WT mice was examined (WT under HFD and WT under STD). 280 genes were significantly deregulated. As expected, WT mice responded to HFD by activating PPARg, which ultimately induced the series of genes responsible for FFA (free fatty acid) uptake, trafficking, TG synthesis by the 2-acylglycerol O-acyltransferase1 (Mogat1) pathway and transport (S10 Fig in S1 Appendix).

To understand the effect of HFD on ABHD11 mutation, the gene expression profile between KO under HFD against KO under STD was compared. Interestingly, no difference in the overall gene expression profile was observed. The genes described for fat phenotype (WT under HFD) were carefully examined in KO under HFD. Genes responsible for fat phenotype are expressed at similar level in KO mice under STD and in KO mice under HFD, indicating that PPARg mediated FFA uptake and TG synthesis and transport pathways were not upregulated in KO under HFD mice.

#### AskOmics analysis of integrated serum metabolomics and liver transcriptomics data

**Fig 6:**
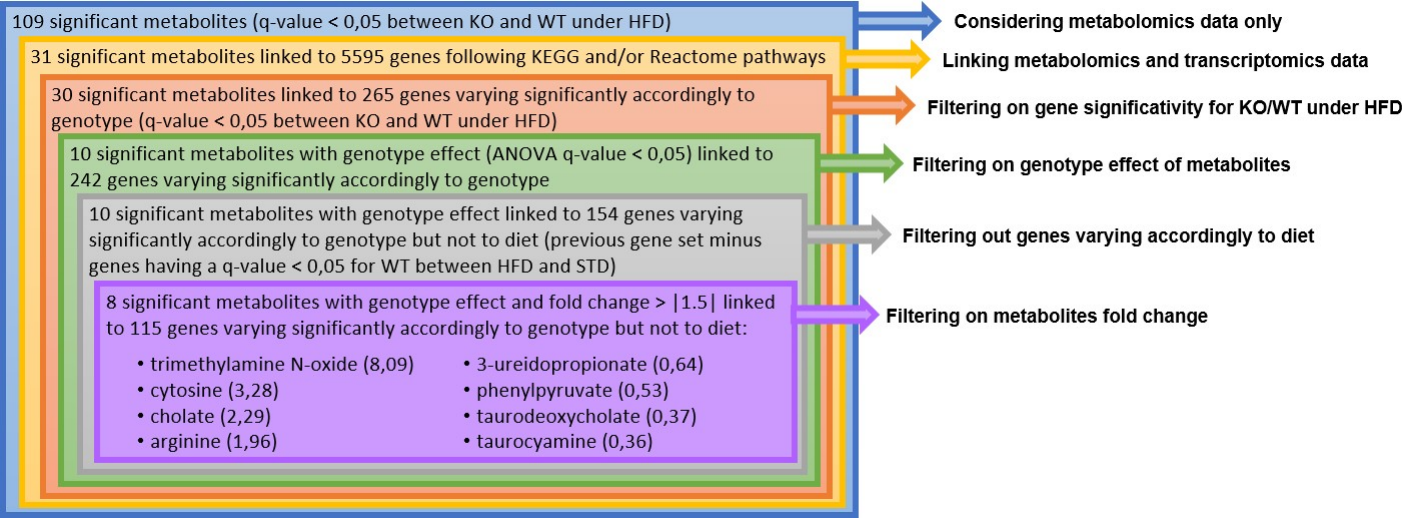
Querying and filtering process to select the most significantly varying metabolites at week 30, between ABHD11 KO and WT mice under HFD linked to genes varying themselves significantly. Values between brachets correspond to fold change ratio (KO/WT).

S11 Fig in S1 Appendix specifies the data-driven model integrated in Askomics. Metabolomic and transcriptomic data were linked using KEGG and Reactome databases. Fig 6 describes the filtering process on KO/WT under HFD data at Week 30. Before the last step, no threshold was applied on fold changes. Considering only metabolomic data, 109 metabolites were found significantly modulated. Among them, 31 were identified as varying significantly and were linked to 5595 genes. Among them, 30 were linked to 265 genes that varied significantly themselves in expression levels between the same groups. For 10 of these metabolites, the ANOVA highlighted a significant genotype effect. Metabolites can be linked to genes varying because of diet or genotype. Filtering out genes varying exclusively because of diet, 154 genes remained, linked to the same 10 metabolites. Finally, applying a fold change threshold of +/- 1.5 on these metabolites, we kept eight of them. Corresponding metabolites and genes data are displayed in S2 Table in S2 Appendix. Five of these eight metabolites are related to the bile acid metabolism.

**Fig 7:**
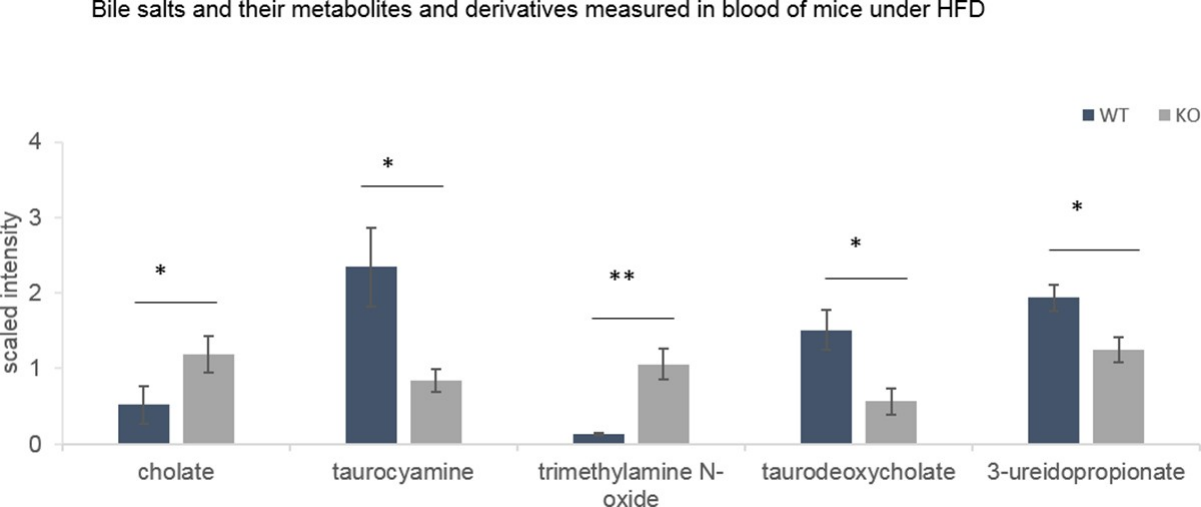
Blood bile salts and their precursors and derivative compounds measured during DIO 2 (30^th^ week). Data from MS analysis (Metabolon analysis) expressed as mean +/-SEM. Two way ANOVA statistical analysis *: qvalue<0.05, **:qvalue<0.01.

Among them, cholate is a primary bile salt (fold change KO/WT = 2.3), taurocyamine is a by-product of taurine which is involved in the conjugation of primary bile salts (fold change KO/WT = 0.4), trimethylamine N-oxide is involved in the cholesterol metabolism that initiates the primary bile salts biosynthesis (fold change KO/WT = 8.1), taurodeoxycholate is a secondary bile salt (fold change KO/WT = 0.4), and 3-ureidopropionate, is involved in the metabolic pathway of the secondary bile salt ursodeoxycholate (fold change KO/WT = 0.6) (Fig 7).

#### qPCR analysis of bile acid transporter and regulator genes in ileum

To evaluate the expression of genes involved in fatty acid binding, transport and esterification (FATP4, CD36, FABP6, FABP2, ACSL3, ACSL5, MGAT2, MGAT3, DGAT1, DGAT2), bile salt transport and regulation (SLC51A, SLC51B, SLC10A2, FGF15, FGFR4), cholesterol transport and regulation (ABCA1, CYP8B1, CYP27A1, HSD17B10), RT-PCR was performed on RNA samples isolated from ileum samples. Among these genes, we observed a slight but significant down modulation of FABP6 and FGF15 in KO mice compared to WT mice under HFD (S12 Fig, in S1 Appendix). These genes are associated with bile salts regulation.

FABP6 was also measured at the protein level by Western Blot in mice ileum samples. Under HFD, the FABP6 protein level was lower in KO ABHD11 mice (S13 Fig, in S1 Appendix).

#### Feces analysis

**Fig 8:**
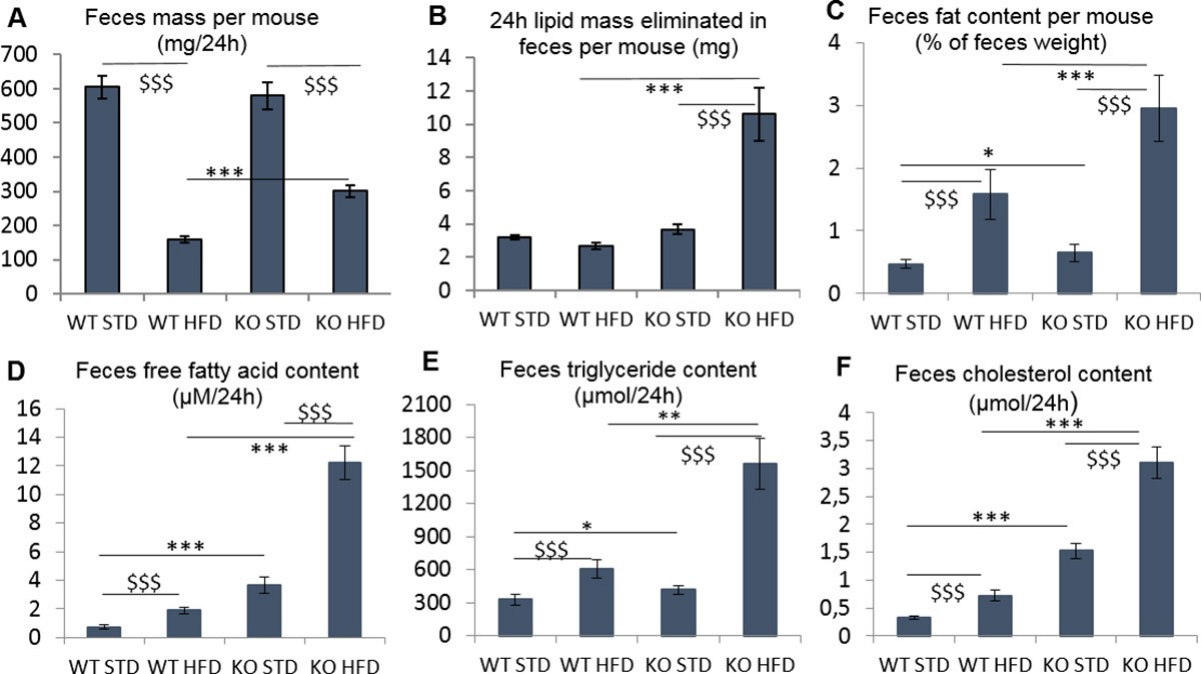
Feces analysis during DIO3 experiment performed at 20^th^ week for 24h. N = 26–27 mice per group. **A-** Feces weight per mouse **B, C-** Lipids extracted from feces per mouse in percentage (B) in mg (C) **D-** Free fatty acid contained in feces **E-** Triglycerides contained in feces **F-** Cholesterol contained in feces. Expressed as mean+/-SEM except graph B expressed as median+/-MAD. Two-way Anova analysis were performed on raw data for graph A, rank transformed data for graph B, log transformed data for graph C, D, E and F. *: pvalue<0.05, **:pvalue<0.01, ***:pvalue<0.001: to compare WT versus KO group in HFD or STD group after a two-way ANOVA analysis. $: pvalue<0.05, $$:pvalue<0.01, $$$:pvalue<0.001: to compare HFD versus STD group in WT or KO genotype after a two-way ANOVA analysis.

As shown in Fig 8A, under HFD, ABHD11 KO mice produced more feces than WT mice despite comparable food consumption and similar energy expenditure. Moreover, the fat content of feces from KO mice under HFD was higher than that from WT mice indicating a larger excretion of lipids in mutant mice (Fig 8B and 8C).

As shown in Fig 8D, 8E and 8F, feces from KO mice under HFD contained significantly higher concentrations of free fatty acids, cholesterol and triglycerides, compared to WT mice, suggesting higher elimination of these two lipids and triglycerides in KO mice. These increases were around 6.5- fold for free fatty acid, 4.3-fold for cholesterol and 2.6-fold for triglycerides.

Under STD, feces from KO mice contained higher concentration of free fatty acid, cholesterol and triglycerides compared to feces from WT mice, but the increase of content was less important than under HFD, respectively 4.9-, 4.75- and 1.3-fold.

Overall, under HFD, KO mice excreted more lipids and triglycerides than WT. Therefore, we conclude that ABHD11 could play a role in intestinal lipid absorption, especially when mice are under HFD.

## Discussion

ABHD11 has been purified from pig brain and identified as a serine hydrolase that can catalyze *in vitro* the hydrolysis of diacylglycerols, especially the 1-stearoyl-2-arachidonoyl-sn-glycerol leading to the synthesis of the endocannabinoid, 2-AG, the most abundant endocannabinoid in tissues [32]. This enzymatic activity was confirmed using purified recombinant human protein. This protein showed very similar biochemical properties to the microsomal 29 kDa DAG lipase described by Farooqui et al., [15] and Rosenberger et al. [20], including the kinetic constants of the enzyme towards the 1-stearoyl-2-arachidonoyl-sn-glycerol.

We confirmed that this alpha beta hydrolase domain-containing-protein is a serine hydrolase implicated in lipid metabolism, as shown by our *in silico* analysis of the primary sequence, revealing the presence of a conserved lipase motif, GXSXG and the Ser, Asp, His catalytic triad. This was also demonstrated in previous studies with heterologous expression of the human ABHD11 in yeast cells [33], or by activity-based protein profiling with different fluorophosphonates derivatives [29, 31, 34].

We have confirmed ABHD11s mitochondrial expression, both in different human cancer cell lines, but also in mouse brain, as previously described by Lefort et al. [35] or Navia-Paldanius et al. [31].

Although different alternative pathways involving PIP2 phosphatase, phospholipase A1, Lysophospholipase C [36] or DAG lipase like DDHD2 [37] may provide 2-AG, DAGLs-α and - β are still considered as the most important enzyme for its biosynthesis. Similarly to both DAGLs, ABHD11 is expressed in vertebrates (human, mouse) but also in other eukaryotes like yeasts or protists [38]. It also shows different tissular and subcellular expression patterns [39], [16], [40], suggesting different physiological roles.

We confirmed previous results obtained by Farooqui et al. [15] in bovine brain extracts that a microsomal 29 kDa from pig brain can produce 2-AG *ex vivo* but we were not able to confirm this *in vivo* by measuring endogenous endocannabinoid levels in brain and in different peripheral tissues from KO and WT mice in homeostatic conditions. A compensation mechanism of 2-AG synthesis cannot be ruled out at this point by other lipases including MAGL. It could also be related to our experimental set-up as some conditions are known to influence endocannabinoid synthesis such as fasting for example. This would need further investigations. We do not also observe any functional impact on mitochondrial respiration. Some specific physiological conditions or stimulations may be necessary to produce 2-AG in mitochondria *in vivo*.

Interestingly, even though ABHD11 is expressed in most tissues and in mitochondria, an organelle playing a key role in cell metabolism, KO ABHD11 mice are viable and present few differences with their littermates even if they show some vision impairment, presumably associated with embryonic development. In terms of body weight under standard diet, only a slight difference is observed and this is exclusively seen in males.

We observed a strong resistance to weight gain of KO ABHD11 mice in DIO studies with associated normal biochemical blood parameters. Transcriptomic analysis performed on the mice livers confirm the absence of diet-induced obesity and metabolic deregulation in animals with deleted ABHD11 gene, suggesting a role of ABHD11 in lipid absorption in the gut. The absence of a differential hepatic expression of genes involved in fatty acid uptake, as well as triglyceride synthesis and transport pathways in ABHD11 KO mice under HFD, indicates that lipids from the diet could be metabolized and/or eliminated before entering into the circulation. Indeed, in mice under HFD, we were able to demonstrate that lipid elimination by feces was more elevated in KO than WT mice, both due to increased fecal matter and lipid content.

A slight decrease in the expression of FABP6 and FGF15 was measured in the ileum of KO mice by RT PCR and by WB for FABP6. Even if this effect was weak and concerned few genes, it could be a signature of a link between ABHD11 and regulation of bile acids. FABP6 is a protein expressed in the ileum able to bind bile acids and to transport fatty acids. It is involved in the entero-hepatic circulation of conjugated bile acids [41] [42]. Primary bile acids are synthetized from cholesterol in the liver, where they are conjugated with taurine or glycine, forming bile salts [43]. After a meal, these bile salts are released into the gastrointestinal tract. Secondary bile salts result from bacterial action in the colon [44]. With the metabolome harvested from the serum of KO mice, we have only an indirect overview of what can happen in the liver and gut of these mice. However, the serum concentration of bile salts reflects 95% of the quantity of these salts present in the intestinal lumen (5% are excreted) [45]. In the blood of KO mice under the DIO study, we detected a modulation in primary and secondary bile salts or precursors or derivatives thereof. The increased concentration of cholic salt, which is upstream from other bile salts biosynthesis pathways, could result from an accumulation as it is transformed to lesser degree in secondary bile salts. We can notice that the significant changes in bile salt concentration observed in our data, could indicate fat absorption impairment in the intestine. This regulation of bile salts in the intestine and its impact on fat elimination in the feces could result from the ABHD11 depletion. It could be interesting to carry out an analysis on the liver genes list that we have shown to be associated with these bile salts, precursors or derivatives.

A putative intra-mitochondrial endocannabinoid system has been described [46, 47] with some data, suggesting regulatory roles of endocannabinoids or 2-AG on mitochondria activity [16, 40, 48, 49]. With the present data, we could not correlate the DAGL-like activity of ABHD11 and its ability to synthesize 2-AG *in vitro* with the strong resistance of ABHD11 KO animal to DIO. We have not demonstrated that the observed phenotype is associated with enzymatic activity of ABHD11. Additional data would be necessary to link the *in vitro* phenotype to the *in vivo* phenotype, focusing on ileum tissues.

Taken together, these data demonstrate a strong role of ABHD11 in weight gain independent from food intake, which likely occurs through impairment of gut lipid absorption. More studies are required to further investigate mitochondrial ABHD11-modulated signaling and metabolic pathways in intestinal tissues to confirm this hypothesis.

## Conclusion

Metabolomics data integrated with the transcriptomics data, show that the resistance of the ABHD11 KO mice to weight gain could come from an impaired ability of the gut to absorb fat. This deficiency would appear to be associated with a modulation of bile salts. Additional experiments would be required to unravel the precise molecular and cellular roles of ABHD11 in weight regulation. The viability of the KO mice combined with their phenotype and molecular features suggest that modulation of ABHD11 or of its signaling pathway could provide an attractive avenue to engage into drug screening and develop new therapeutic agents for metabolic disorders.

## Supporting information

S1 Appendix (DOCX)

S2 Appendix (XLSX)

S3 Appendix (PDF)
