# Supplementary material for: ABHD11, a new diacylglycerol lipase involved in weight gain regulation
Source: PLoS One. 2020 Jun 24;15(6):e0234780. doi: 10.1371/journal.pone.0234780 (PMC7313976; doi:10.1371/journal.pone.0234780)
Supplement: S1 Appendix — (DOCX) [file pone.0234780.s001.docx]

**Appendix A – supporting Information - Figures**


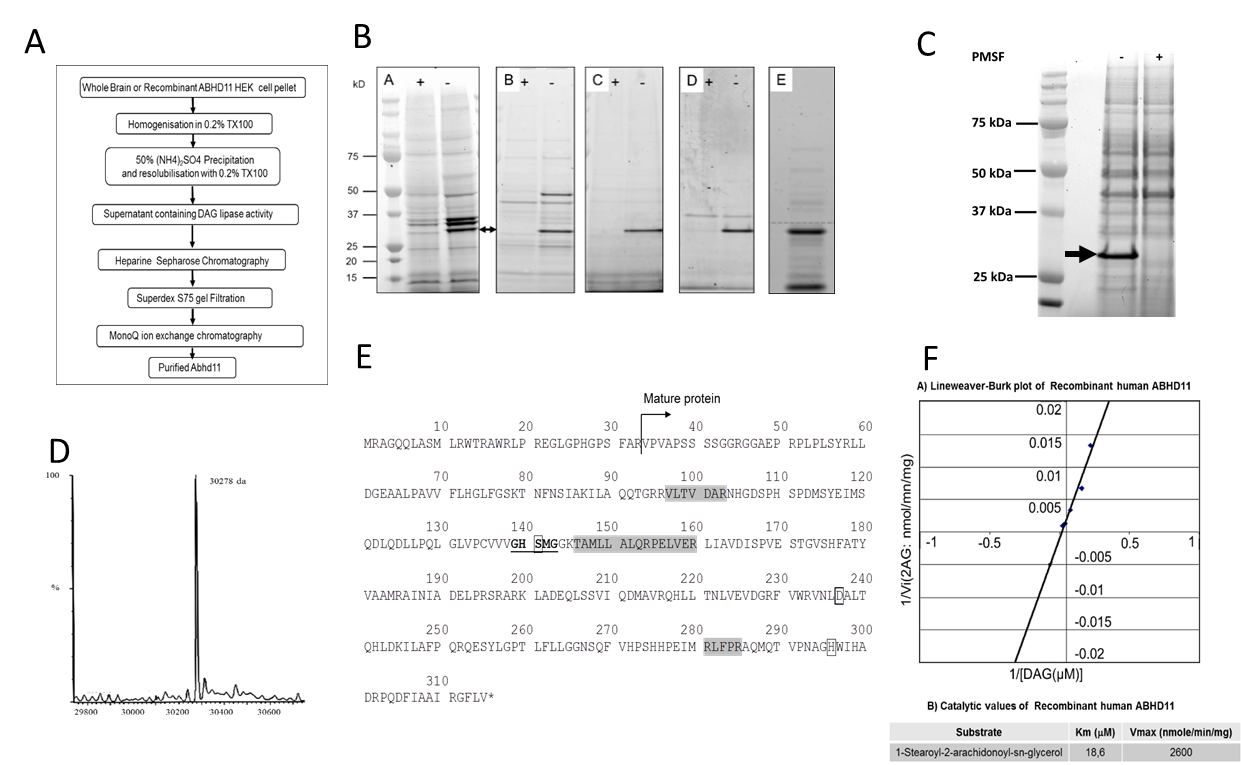


**Figure S1: ABHD11 purification from pig brain or human recombinant HEK293 and characterization**

**A-** Global process of ABHD11 purification from pig brain or human recombinant ABHD11 expressed in HEK293.

**B-** ABPP profile following purification steps from pig brain. Labels + & - indicate PMSF (+) or vehicle (-). (A) Triton TX100 whole pig brain extract showing a 29 kDa band (arrow) which is inhibited by PMSF (+) and represents the potential DAG lipase targeted for purification. The two upper bands indicate monoacylglycerol lipase (MGL), another serine hydrolase, confirmed by Western blot analyses (Data not shown). (B) Pool after Heparin Sepharose step. (C) Pool from S75 superdex Gel filtration. The selected pool around 30 kDa contains the targeted protein. (D) Detection by ABPP of 29Kd proteins eluted from MonoQ ion exchange chromatography indicating a purification of DAG activity to homogeneity which can be associated to a protein as shown with Sypro ruby staining (E). **C-** ABPP profile of human recombinant ABHD11 following purification step on Heparin-Sepharose. TX100 extracts from HEK293 cells transfected by ABHD11 were purified on Heparin-Sepharose and submitted to ABPP. Labels +/- indicate PMSF 1mM (+) or vehicle (-). The transfected hABHD11 eluted from Heparin by 0.5M NaCl was detected (Black arrow) only in the absence of PMSF. **D-** MS profile of purified human recombinant ABHD11. **E-** Primary Structure of human ABHD11. Highlighted in grey the tryptic peptides identified from pig brain. GXSXG Lipase motif (by similarity) underlined. Serine hydrolase triad motif (by similarity) in boxes. Mitochondrial signal peptide: amino acids 1 to 33, mature protein N terminal sequence indicated with the arrow. **F-** Catalytic values of purified human recombinant ABHD11

1. Lineweaver-Burk plot showing 2-AG formation with varying concentrations of ABHD11.
2. mean catalytic constants following incubation with 1-Stearoyl-2-arachidonoyl-sn-glycerol.


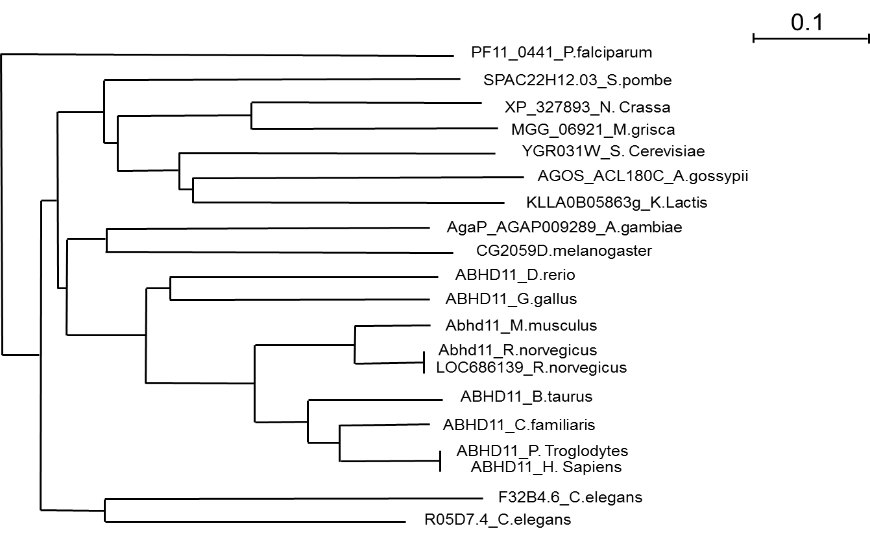


A

B


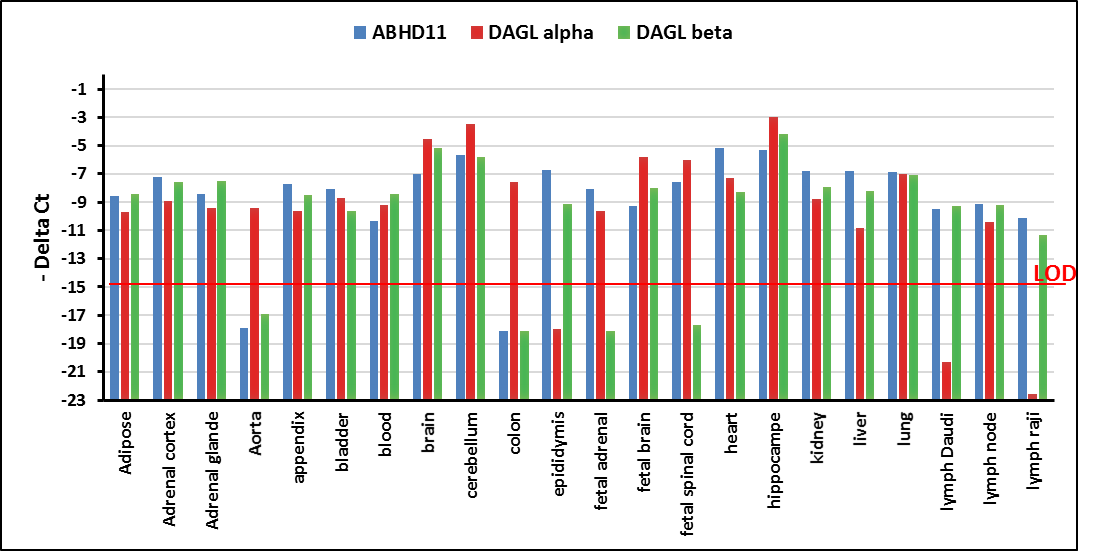

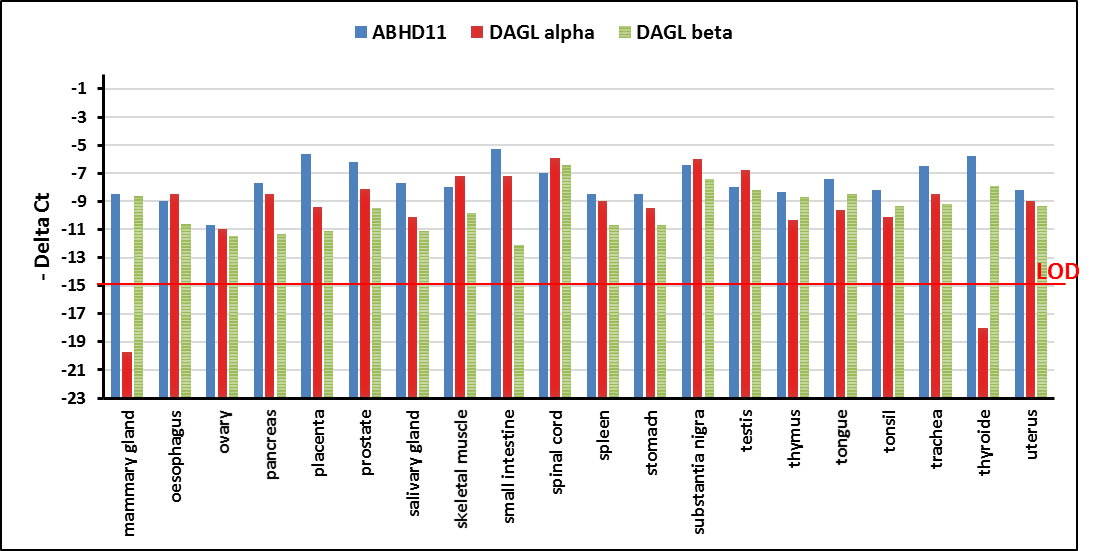


C

**Figure S2: Expression profile of ABHD11. A-** protein sequence alignment between human ABDH11 (Q8NFV4), human DAGLA (Q9Y4D2) and human DAGLB (Q8NCG7). ABHD11 exhibits 6.2% identity with DAGLA, and 9.0% identity with DAGLB. **B-** Dendogram profiling of human ABHD1 1 and various orthologs of ABHD11, representing several different species. The nomenclature used on the figure is "Gene_Species" where "Gene" is either the gene name found in the NCBI gene file or the RefSeq identifier when no gene name was found, and "Species" is the first letter of the genus, followed by the name of the species as taken from the Refseq sequence definition line. **C-** gene expression profile of ABHD11 in human tissues. Expression of the following genes was studied for each tissue: ABHD11 (blue), DAG-alpha (red), DAG-beta (green).

**
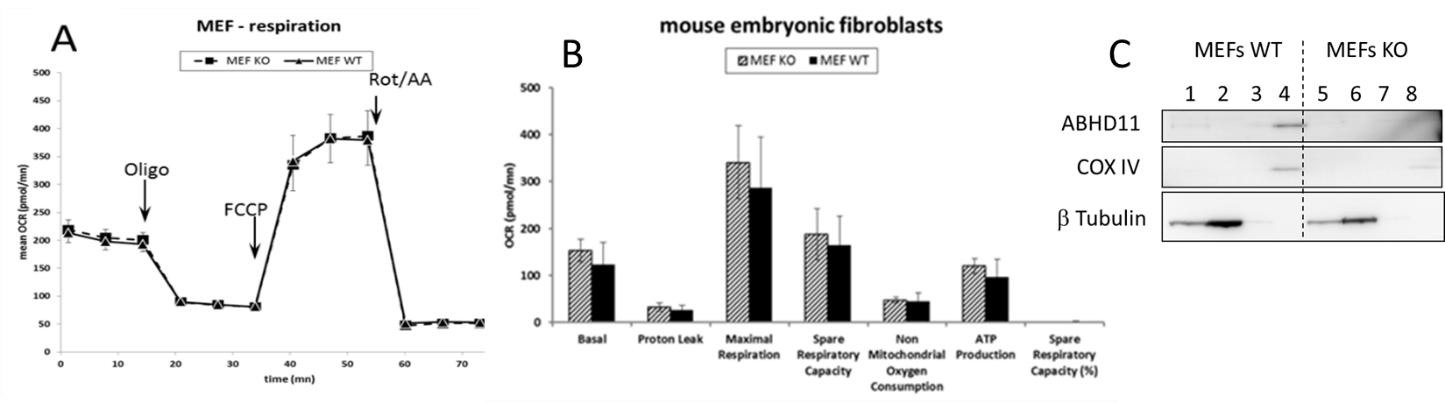
**

**Figure S3:** **Mitochondrial respiration in mouse embryonic fibroblasts**. Comparison between ABHD11-WT and KO Mouse Embryonic Fibroblasts respiration. Data are expressed as mean +/-SD. **A-** Respiration profile from eight preparations of MEFs ABHD11 WT (triangles, hard line) and of MEFs ABHD11 KO (squares, dash line). OCR measured after injection of 1.5µM Oligomycin, 1µM FCCP and 0.5µM Rotenone/ AntimycinA in time course were superimposed. **B-** parameters of mitochondrial function calculated with XF mitostress test report generator, mean of 8 experiments. **C**- Western blot of ABHD11 expression in MEFs mitochondria fraction from WT and KO mice: MEFs lysates (1, 5) were concentrated with Qiagen mitochondria isolation kit and cytosol fractions (2, 6), mitochondrial fractions before (3, 7) and after concentration (4, 8) and all these fractions were analyzed by Western blot.


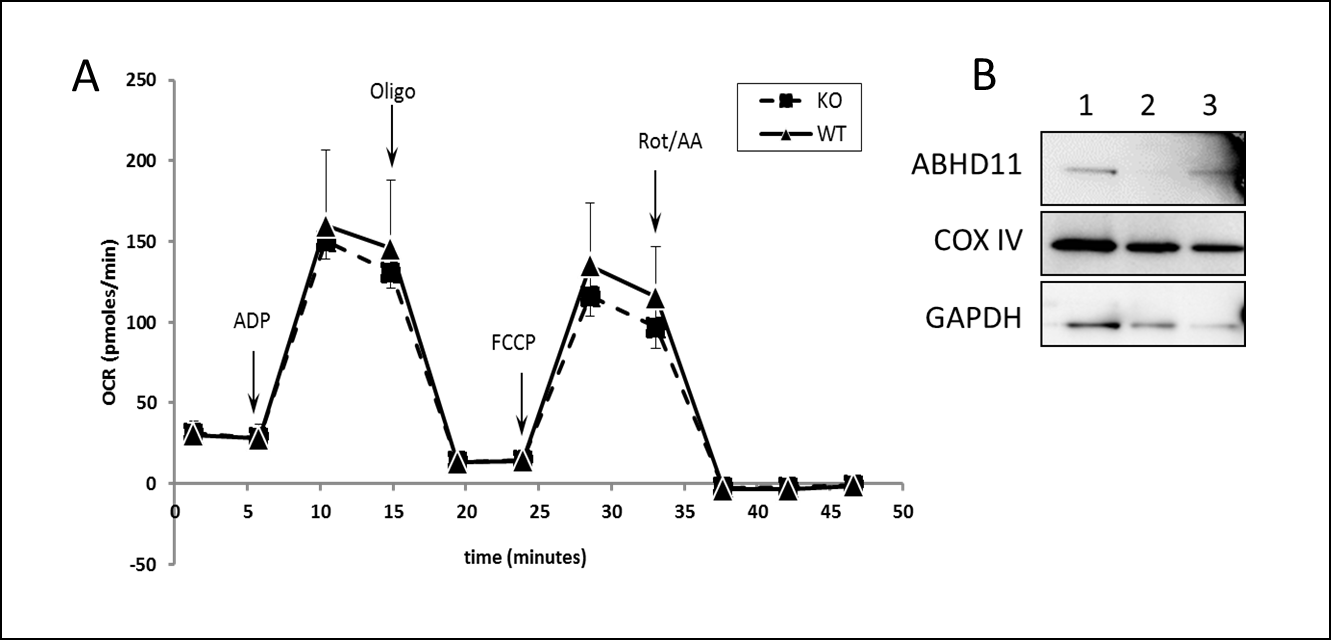


**Figure S4: Mitochondrial respiration in liver isolated mitochondria**. A- Seahorse XFe96 measurements of oxygen consumption (OCR) of isolated mitochondria from liver of wild type and knock out ABHD11 mice *(n=4 WT; n=4 KO)*. Comparison between mitochondrial respiration profiles obtained after addition of 4 mM ADP, 3 µM Oligomycin, 4 µM FCCP and 4 µM Rotenone/ Antimycin A, mean of experiments. Data are expressed as mean +/-SD. B- Western blot of ABHD11 expression in liver isolated mitochondria from KO (2) and WT (3) mice compared to mitochondrial fraction of MEFs WT in figure S3 lane 4 (1).


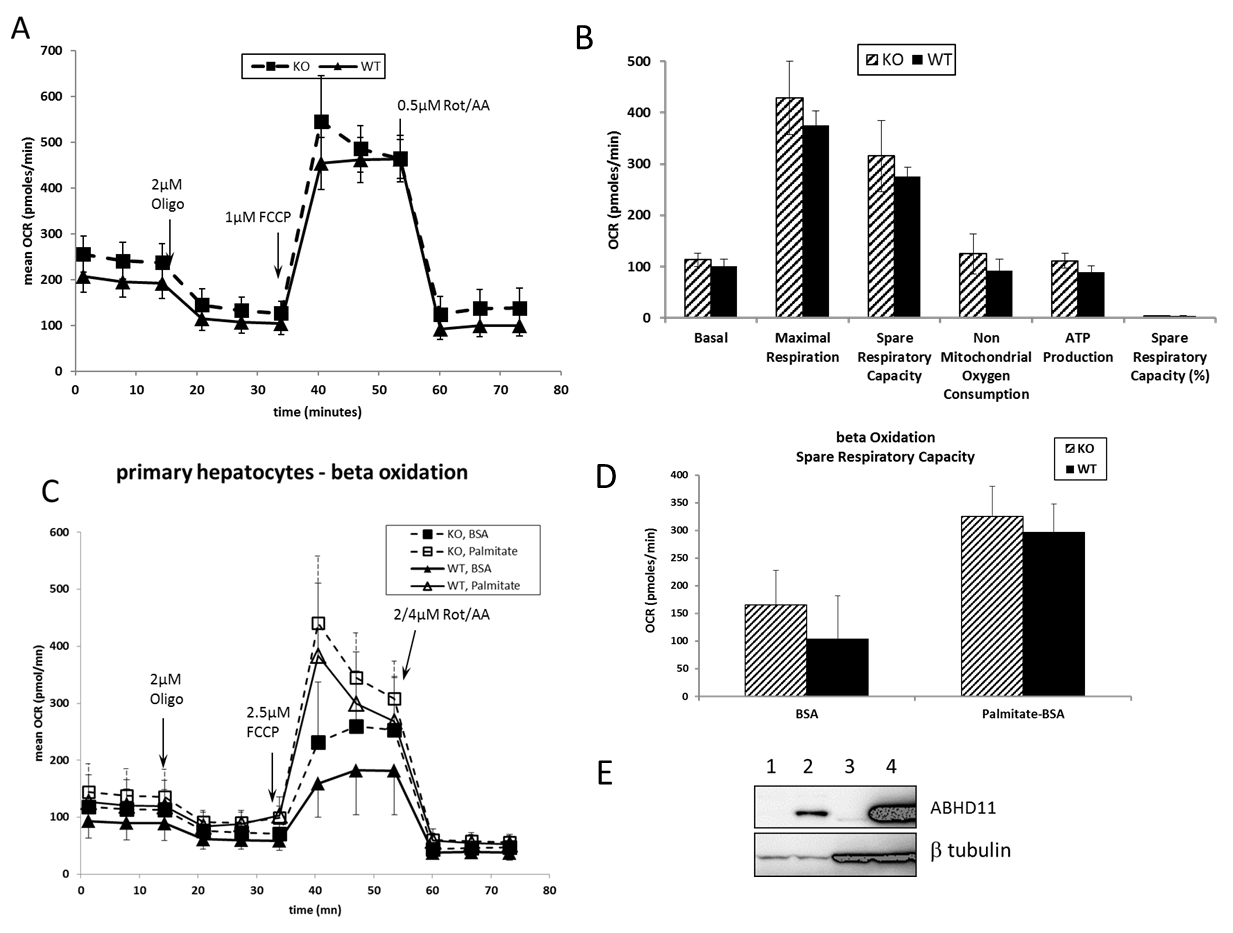


**Figure S5 : mouse primary hepatocytes. A-B-** Seahorse XFe96 measurements of oxygen consumption (OCR) of freshly prepared primary hepatocytes from wild type and knock out ABHD11 mice *(n=5 WT; n=4 KO)*. mitochondrial respiration profile (A). Parameters of mitochondrial function calculated with XF mitostress test report generator (B). Measurements after addition of 2µM Oligomycin, 1µM FCCP, 0.5µM Rotenone/AntimycinA. Data are expressed as mean +/-SD. **C-D-** Seahorse XFe96 measurements of oxygen consumption (OCR) in beta oxidation conditions of freshly prepared primary hepatocytes from wild type and knock out ABHD11 mice *(n=4 WT; n=4 KO)*. Palmitate-BSA or BSA were added just before respiration measurements. Respiration profile of primary hepatocytes in the presence of palmitate or BSA (C). Comparison of percentage of spare capacity (= (maximal respiration / basal respiration) x100) (D). Measurements after addition of 2µM Oligomycin, 2.5µM FCCP and 2µM Rotenone + 4µM AntimycinA. Because of the high difference in basal and maximal respiration through experiments, spare capacity parameter was the most appropriate for comparison. Data are expressed as mean +/-SD. **E**- ABHD11 protein detection by Western Blot in mouse primary hepatocytes from liver of KO mouse (1) or WT mouse (2) compared to HEK293 control (3) and HEK293 transfected by mouse ABHD11 (4).

**D**


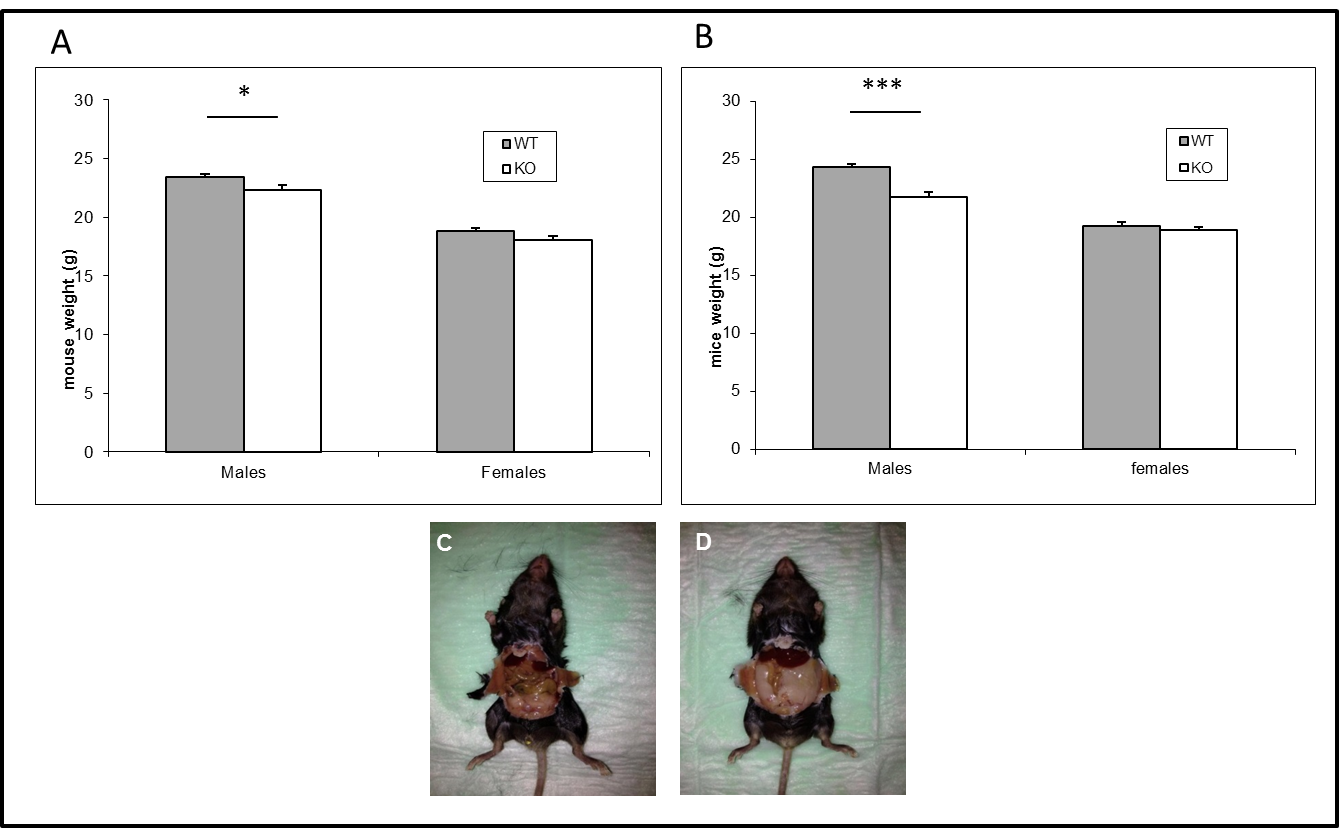


**Figure S6: KO/WT ABHD11 mice phenotyping. A-** 6 weeks old mice weight **B-** 9 weeks old mice weight. Represented data are mean +/- SEM (* p=0,023, *** p<0,0001 WT vs KO). N=11 KO male, N=9 KO female, N=13 WT male, N=13 WT female. **C, D-** Abdominal fat in male at 19 weeks of age in KO mouse (C) and in WT mouse (D), weighing respectively 26 and 32g. These images are representative of all observed mice. This difference is not present in female mice.

**Figure S7: Biochemical analyses and behaviour tests (SHIRPA) with males ABHD11 WT and KO mice at 9 week of age.** Data are expressed as mean+/-SD. **A-** Blood biochemical analyses **B-** Locomotor test **C-** Muscular strength test (Grip test) ratio Grip means (3 trials) / mouse weight **D-** vision test, **E-** Touch escape test (reactivity)


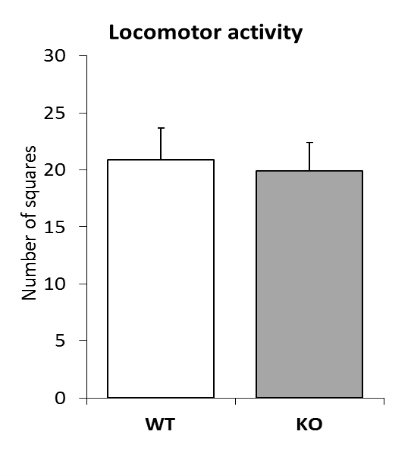


B


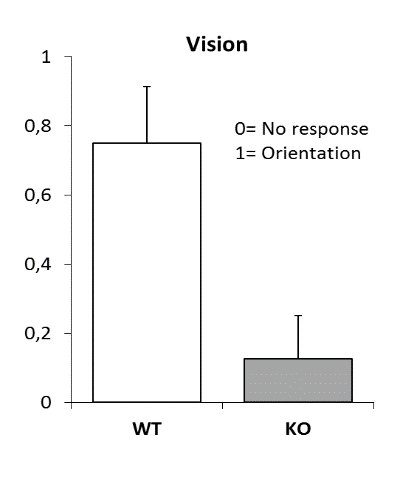


D


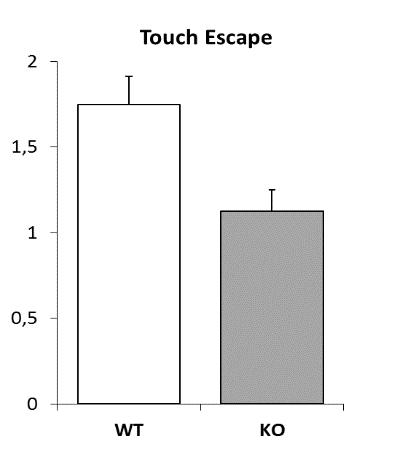


E


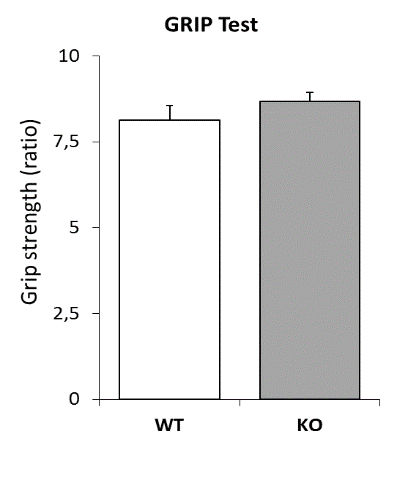


C


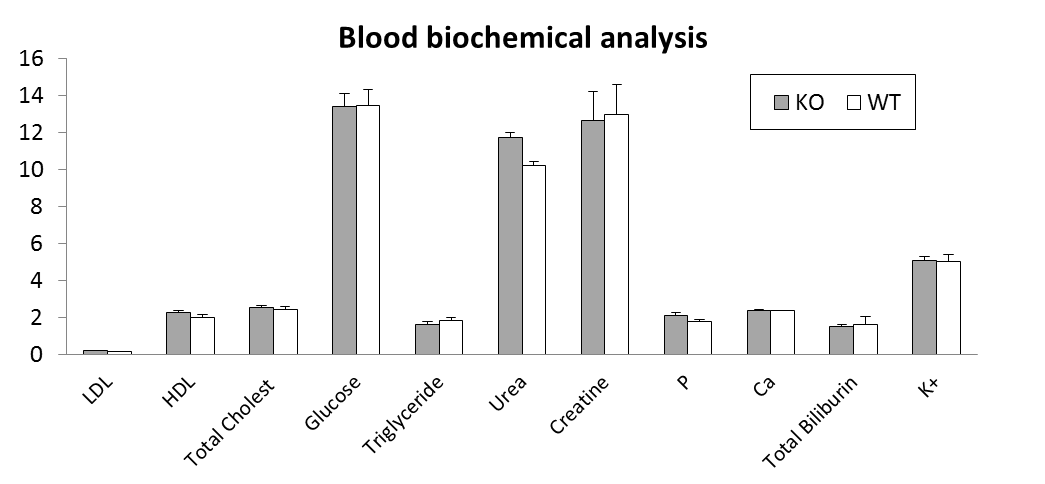


A


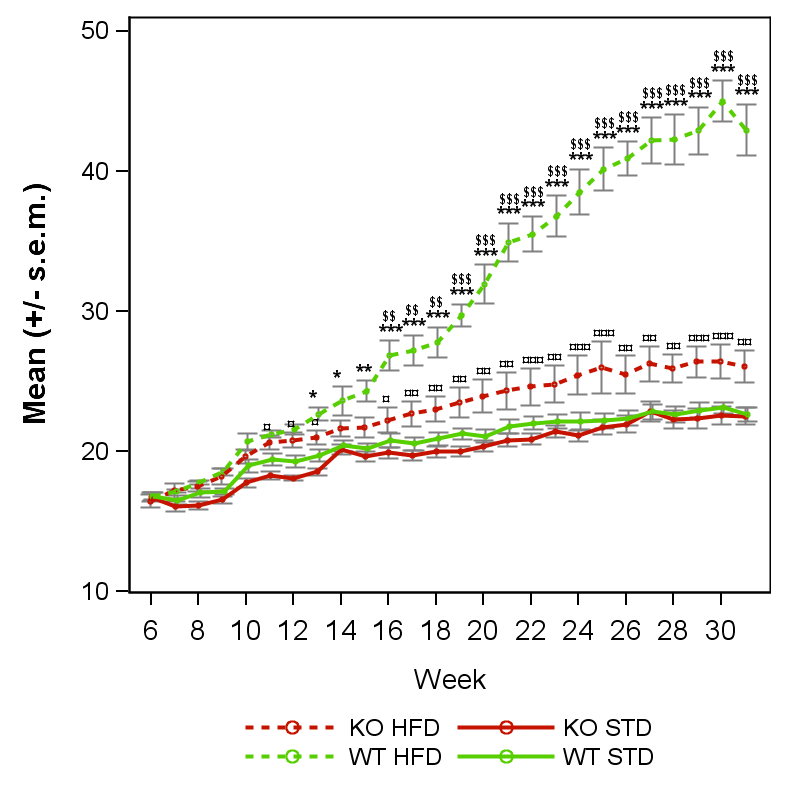

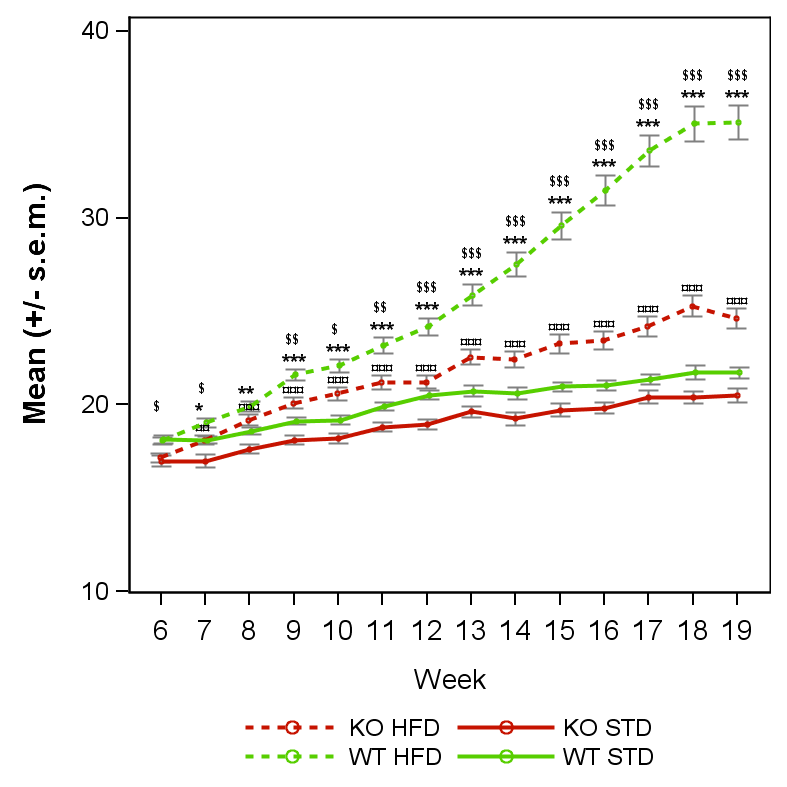


**B**- DIO3 females

**A**- DIO2 females


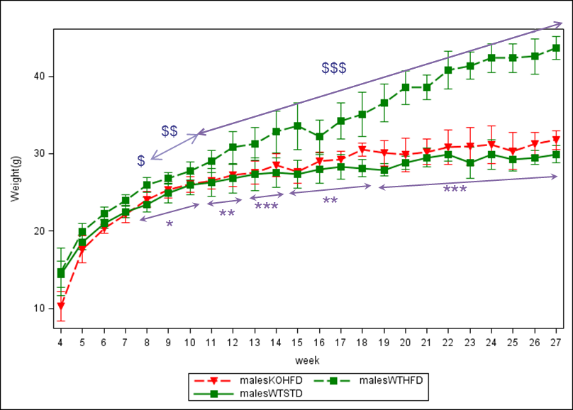


**C**-

**D**- DIO1 males

**Figure S8: supplemental data for DIO 1, 2 and 3**

**A-B:** Weight evolution (g) by genotype and diet in DIO2 study (A) and DIO3 study (B). The curves represent means +/- s.e.m. at each day for each group (n=8 for DIO2 and n=27 for DIO3). *: pvalue <0.05, **: pvalue <0.01, ***: pvalue <0.001; obtained using a post-hoc analysis to compare HFD versus STD group in WT genotype after a three-way ANOVA with repeated measures on time factor. **¤**: pvalue <0.05, **¤¤**: pvalue <0.01, **¤¤¤:** pvalue <0.001: obtained using a post-hoc analysis to compare HFD versus STD group in KO genotype after a three-way ANOVA with repeated measures on time factor. $: pvalue <0.05, $$: pvalue <0.01, $$$: pvalue <0.001: obtained using a post-hoc analysis to compare WT versus KO group in HFD group after a three-way ANOVA with repeated measures on time factor. #: pvalue <0.05, ##: pvalue <0.01, ###: pvalue <0.001: obtained using a post-hoc analysis to compare WT versus KO group in STD group after a three-way ANOVA with repeated measures on time factor. **C**: Cumulative food intake measured during 24h once a week by mouse during DIO3 (n=3 box per group, Data are expressed as mean+/-SD). **D**: Weight evolution (g) by genotype and diet in male mice from DIO 1 study. The curves represent means +/- MAD at each day for each group (n=8). *: pvalue <0.05, **: pvalue <0.01, ***: pvalue <0.001; obtained using a post-hoc analysis to compare HFD versus STD group in WT genotype after a three-way ANOVA with repeated measures on time factor. $: pvalue<0.05, $$:pvalue<0.01, $$$:pvalue<0.001: obtained using a post-hoc analysis to compare WT versus KO group in HFD group after a two-way ANOVA with repeated measures on time factor


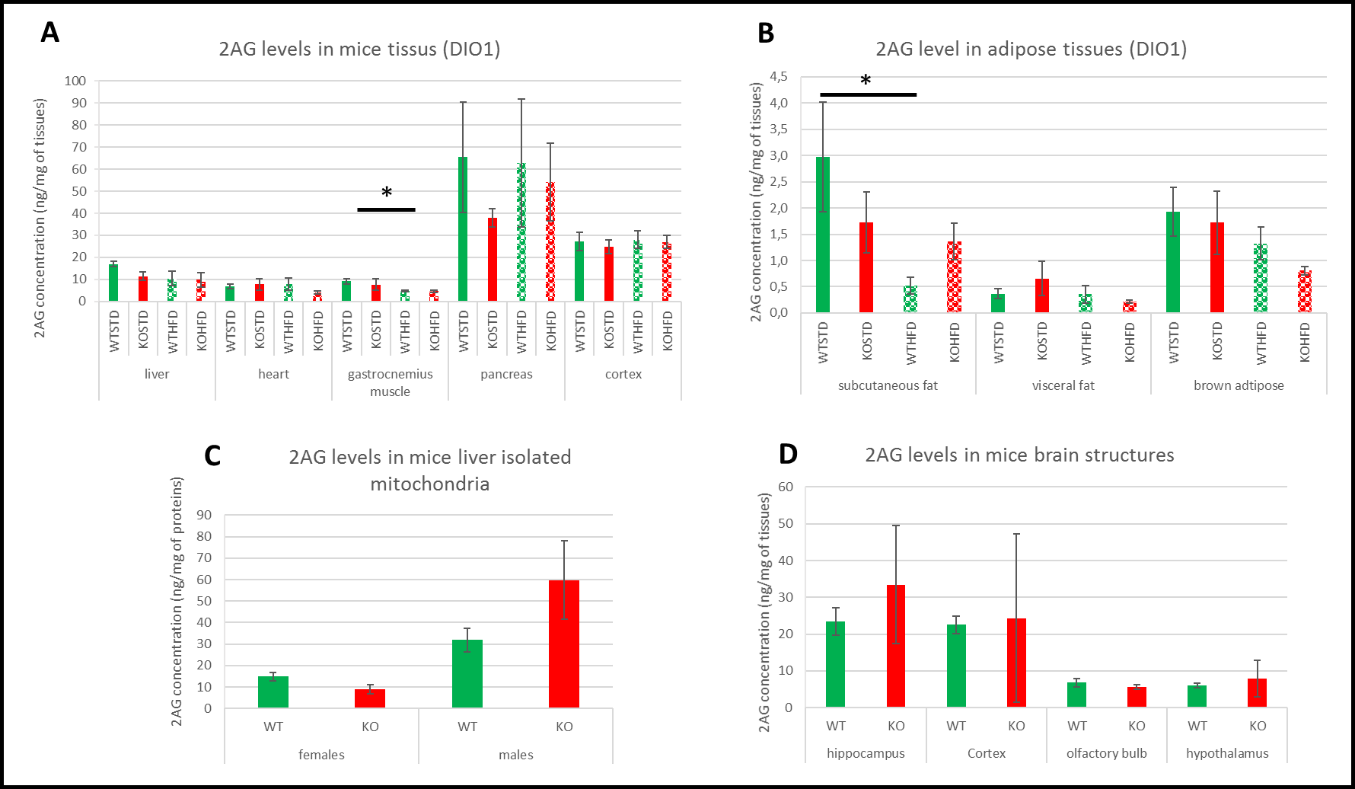


**Figure S9: 2AG levels in mice tissue**

**A-B-** 2AG levels measured at the end of DIO1 (6-7 mice/group) **C-** 2AG levels measured in liver isolated mitochondria from male and female mice under standard diet (8 mice/group) **D-** 2AG levels measured in brain structures from male mice under standard diet (2-3 mice/group). Represented data are mean +/- SEM for graphs A, B, C and mean +/- SD for graph D. Two ways ANOVA statistical analysis for graphs A and B for genotype and diet effect (*: pvalue <0.05). Student T test analysis for graphs C and D, no significant effect.


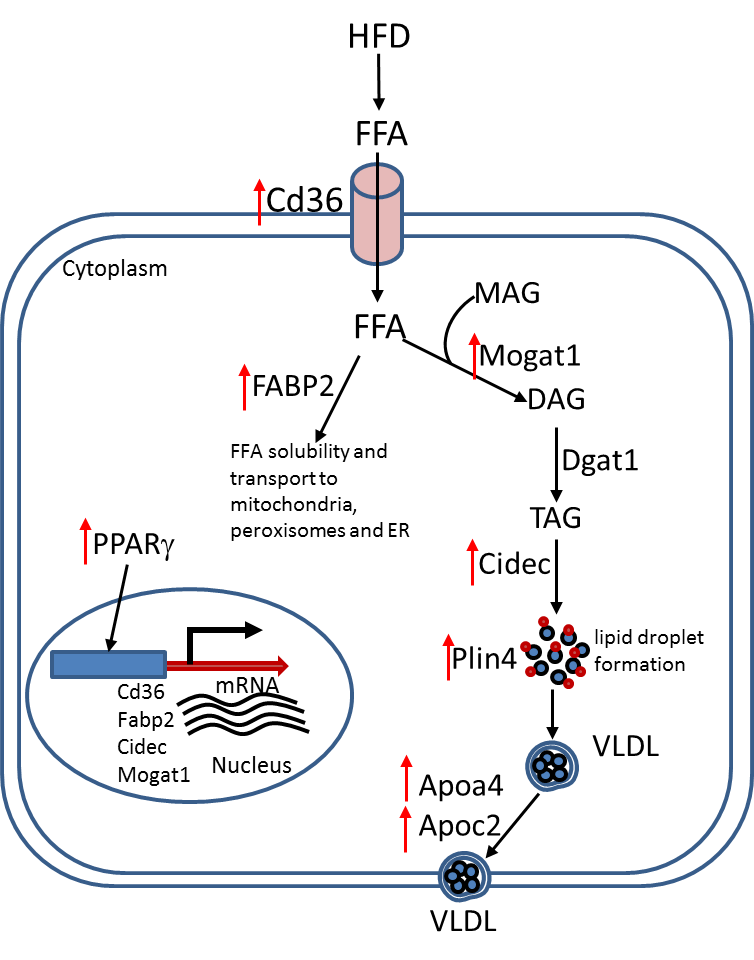


**Figure S10. PPARγ mediated FFA metabolism in liver samples from WTHFD.** WT mice respond to HFD by activating PPARg which ultimately induced the series of genes responsible FFA uptake, trafficking, TG synthesis by Mogat1 pathway and transport (fold change values are provided in supplementary table S1 appendix B).  When compared with WTSTD, PPARγ pathway genes up regulated WTHFD (indicated by red arrow). Interestingly same set of genes expressed at similar level in KOSTD and KOHFD.


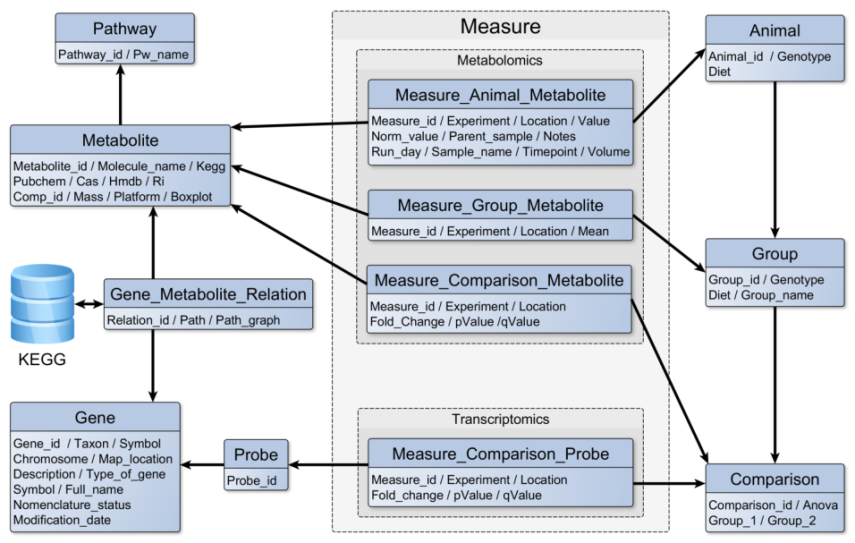


**Figure S11: Data-driven model of the metabolomics and transcriptomics studies results integration**

**
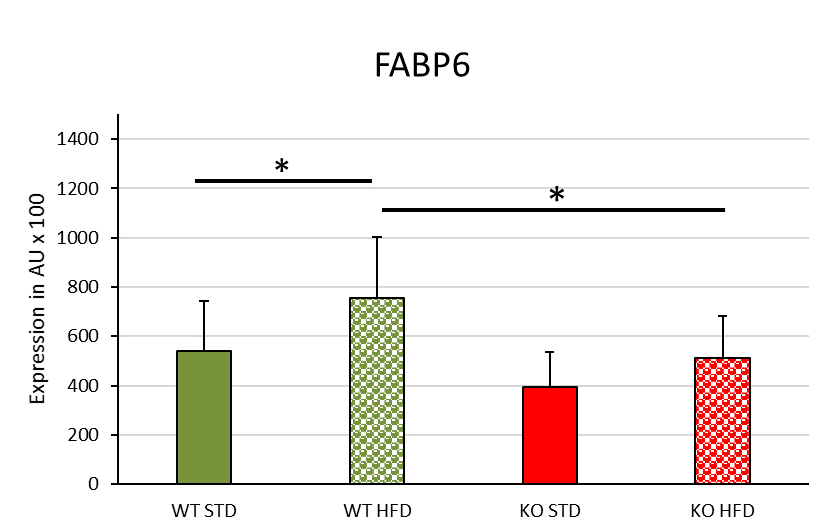

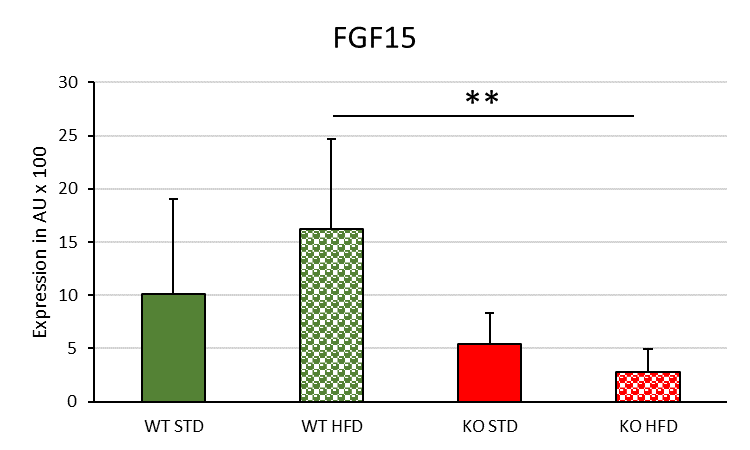
**

**B**

**A**

**Figure S12: Expression level of Fabp6 and FGF15 in the ileum measured at the end of DIO2**

The histograms represent means +/- standard deviation for each group (n=8). **A-** Fold change WT HFD group versus WT STD group = 1.4 (Ct value for WT STD group= 16.8) and fold change KO HFD group versus WT HFD group = 0.68 **B-** Ct value for WT STD group= 23.2 and fold change KO HFD group versus WT HFD group = 0.2. One-way ANOVA statistical analysis (Newman-Keuls). *: pvalue <0.05, ** pvalue <0.01.

**A**

**
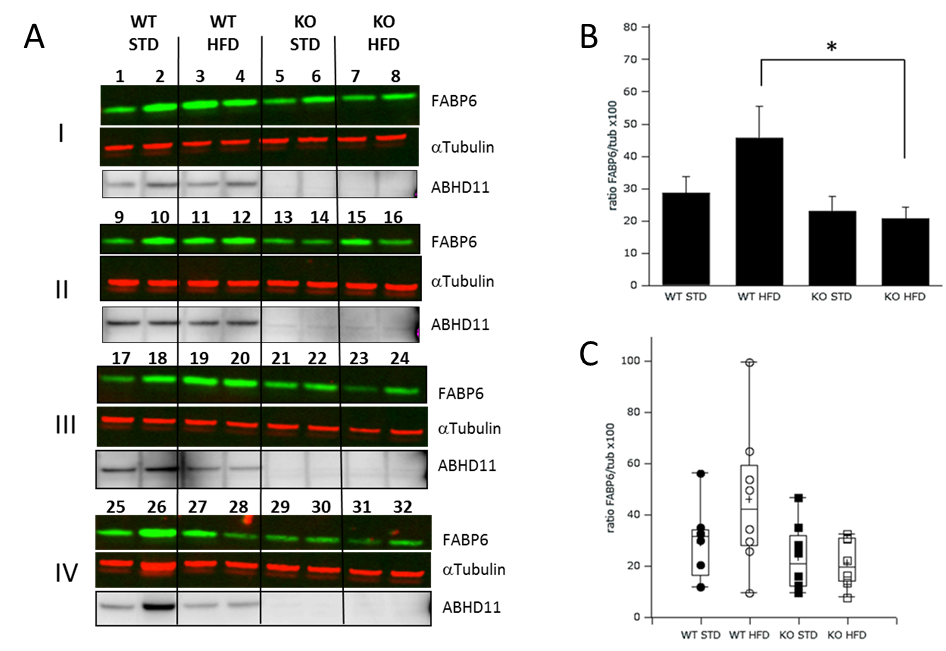
**

**Figure S13: A- Western blot of FABP6 and ABHD11 protein expression in ileum lysates of the 32 mice of DIO3**. Lysates of ileum of each mouse of DIO3 (8 mice in each group) were loaded (20µg) on SDS-PAGE, transferred on nitrocellulose membranes (I, II, III and IV: 2 mice of each group per membrane) and analyzed by Western blot. ABHD11 Ab from SantaCruz, ECL detection. FABP6 Ab from Abcam and a-tubulin from Cell Signaling, infrared detection**. B- C- Quantification of FABP6 protein and comparison between groups:** FABP6 and Tubulin quantification were performed using Image Studio (Li-Cor) and the ratios were calculated: Data are expressed as mean +/-SEM (B) and in Box plot representation (C). Under HFD, the expression of FABP6 is lower in KO mice compared to WT mice (p=0.0087). Two way ANOVA statistical analysis *: pvalue <0.05.


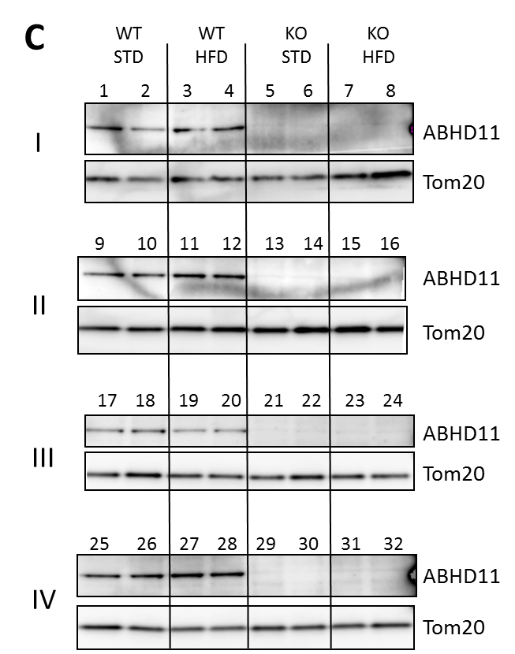


**A**

**B**


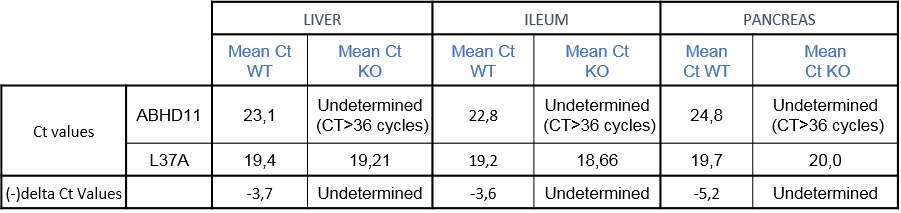


**Figure S14: Validation of the knockout of ABHD11 gene in tissues of transgenic KO ABHD11 mice.**

**A-B**: ABHD11 gene expression level in 3 different tissues in WT and KO mouse model group (8 WT and 8 KO adult female mice). Values are normalized with RPL37a and expressed as -delta Ct (-(Ct ABHD11-Ct RPL37a)). Undetermined= no detected (Ct>36 cycles) in table.

**C**: ABHD11 protein expression. Western blot of ABHD11 in mice liver lysates : Lysates of liver of each mouse at the end of DIO experiment (8 mice in each group) were loaded (20µg) on SDS-PAGE, transferred on nitrocellulose membranes (I, II, III and IV: 2 mice of each group per membrane) and analyzed by Western blot. ABHD11 Ab from SantaCruz, Tom20 Ab from Cell Signaling, ECL detection.

**B- supplemental methods**

# ABHD11 Purification from pig brain

## Extraction from Frozen pig brain

One pig brain was homogenized in homogenizing buffer A (0,1 M MOPS pH 7,4; 0,2% TritonX100) without protease inhibitors in a motor-driven stainless-steel mixer, transferred and left in a glass container for 18 hours, with constant stirring. The suspension was centrifuged at 10000 g for 15 minutes to remove debris. The 200 mL supernatant (SN1) was centrifuged at 100000 g for 1 hour and subjected to (NH4)_2_S04 precipitation. All these steps were carried out at 4 °C.

## (NH4) 2SO4 precipitation

The SN1 supernatant was adjusted from 0 to 30 % (NH4)_2_S04 and equilibrated overnight, then centrifuged at 4°C at 100000 g for 30 minutes in polycarbonate bottles. The pellets after centrifugation were resuspended in buffer A, and the suspension was centrifuged for additional 30 minutes at 100000 g to remove insoluble material. The resulting Triton extract (SN2) was analyzed by ABPP with FP-Bodipy probe and used as starting material for further purification.

## Heparin-Sepharose affinity chromatography

The pooled fractions were then applied to a 5 mL Heparin-Sepharose Hitrap column (GE Healthcare) equilibrated in 0,1 M MOPS buffer pH7,4. The column was washed with this buffer at a flow rate of 1 mL/min and then eluted with a step gradient of 0,5 M NaCI in the same buffer. Fractions of 5 mL were collected and 1 % of each fraction was labeled with FP-Bodipy and analyzed as described next.

## Superdex S-75 Gel Filtration

Soluble extracts from a 30% saturated-(NH4)_2_SO4 sample were loaded on Hiload Superdex-75-26/60 equilibrated in 50 mM MOPS pH 7,4 at a flow rate of 5 mL/min. All the chromatography procedures were performed in a 4°C cold room.

## Reverse Phase HPLC

Reverse phases HPLC purification were done using a C4 reverse phase column (Brownlee Labs cartridge). The column was equilibrated in 70% eluent A (0,1% TFA in H20) and 30% eluent B (CH3CN-0,1% TFA) at 300 μL/min followed by a linear gradient at 50% B in 40 min and monitored at 210 nm. 1,2 ml fractions were collected and 10% of each fraction was dried under vacuum with Laemmli buffer for SDS-PAGE analyses and ABPP profiling.

## ABPP Experiments

Activity based protein profiling (ABPP) reactions with a probe referred to as FP-Bodipy were performed in a 100 μL format in 0,1 M MOPS buffer pH 7,4 of different tissue, cell extracts or purification fractions. Inhibitors were added at the indicated concentration and the mixtures were incubated for 30 min at 4°C. FP-Bodipy was then added at a final concentration of 0,1 μΜ. After 30 minutes incubation at 4°C, the labeling reaction was stopped by adding 50 μL of 2X gel loading buffer and heating at 60°C for 15 min. The samples were then centrifuged and analyzed by PAGE/SDS electrophoresis. Labeled proteins were in-gel visualized by using a flatbed fluorescence scanner (Typhoon-Biorad). Concentration-dependent inhibition curves were obtained from integrated gel band intensities (Quantity one software-BIORAD). Enriched fractions were also treated with 1 mM PMSF or DMSO for 30 minutes and then incubated with a biotinylated FP-biotin probe for 60 minutes. Probe-labeled proteins were enriched and characterized by a combination of avidin affinity and liquid chromatography-mass. The probes referred to as FP-Bodipy and FP-Biotin were identical to the probe shown on Figure 1 of Jessani and Cravatt [1], except that the Tag was bodipy or biotin instead of rhodamine.

# Protein characterization by LC-MS

## Protein digestion

Purified human recombinant ABHD11 was solubilized in 6 M urea and reduced with 10 mM DTT in 25 mM ammonium bicarbonate pH 8.9 at 56 °C for 30 minutes. lodoacetamide was then added to a final concentration of 20 mM. The resulting mixture was incubated at room temperature in darkness for 30 minutes. The mixture was then diluted 6-fold to reduce urea concentration and after addition of trypsin (1:50 protease-to-protein ratio) was incubated overnight at 37°C and submitted to LC-MSMS Orbitrap analyses.

## Nano-LC-MS/MS analysis and data processing

Nano LC-MS/MS experiments were performed on an Ultimate 3500 RSLC dual system coupled to a hybrid LTQ Orbitrap Elite mass spectrometer equipped with a nanoelectrospray source (Thermo Scientific). Tryptic digests were loaded onto a C18 trap column (Acclaim PepMap100) and washed with 0.2% HCOOH at 5 µL/min for 10 min. Peptides were then eluted on a C18 reverse-phase column (Acclaim PepMap RSLC) with a linear gradient of 4-30% solvent B (H2O/CH3CN/HCOOH, 10:90:0.2 volume) for 120 min, 30-90% solvent B for 10 min, and 90% solvent B for 5 min at a flow rate of 250 nL/min. The mass spectrometer was operated in the data-dependent mode to automatically switch between MS and MS/MS acquisition. Survey full scan MS spectra (m/z 300-1,600) were acquired in the Orbitrap with a resolution of 120,000 at m/z 400. The AGC was set to 1 × 106 with a maximum injection time of 200 msec. The ions were then isolated for fragmentation in the LTQ linear ion trap. Most intense ions (up to 20) were fragmented with normalized collision energy of 28 % at the default activation q of 0.25 with an AGC of 1 × 104 and a maximum injection time of 50 msec. The dynamic exclusion time window was set to 50s. LC-MS/MS data, acquired using the Xcalibur software, were processed using a homemade Visual Basic program software developed with XRawfile libraries to generate a MS/MS peak list (MGF file) that is used for database searching. Database searches were done using MASCOT (matrix Science; http://www.matrixscience.com/) with the Swiss-Prot database. The search parameters used for post-translational modifications were fixed modifications of +57.02146 Da (carbamidomethylation) on cysteine residues and dynamic modifications of +15.99491 Da on methionine residues (oxidation) and -17.026549 Da on N-terminal glutamine residues (N-PyroGlu). The precursor mass tolerance was set to 5 ppm and the fragment ion tolerance was set to 0.5 Da. The number of missed cleavage sites for trypsin was set to 2. Mascot result files were imported into Scaffold software. Scaffold (Proteome Software Inc., Portland) was used to validate MS/MS based peptide and protein identifications. Queries were also used for XTandem parallel Database Search. The compiled results of both database searches were exported for analysis.

## MS analysis of purified ABHD11 human recombinant protein

Intact molecular weights were measured by LC/MS using an Ultimate 3500 RSLC dual system coupled to a hybrid LTQ Orbitrap Elite mass spectrometer (Thermo Fisher Scientific) equipped with a nanoelectrospray source. Reverse phase chromatography was performed with a binary buffer system consisting of 0.2% formic acid (buffer A) and 80 % acetonitrile in 0.2 % formic acid (buffer B). About 1 pmol of purified human recombinant protein was loaded on a Poros 1 R/H column (75 µm x 15 cm, Dionex). The protein was eluted by a linear gradient of buffer B (25% to 50 % in 10 min, 50 % to 90 % in 2 min) for a total 35 min gradient run with a flow rate of 250 nL/min. Mass spectra (m/z 500-2,000) were acquired in the positive ITMS mode with 5 µscans accumulation, a target value of 30000 and a maximum injection time of 100 msec. The acquired raw files were converted in MassLynx format (Waters) using a home-made program and then were deconvoluted using MaxEnt software (Waters).

# Cloning and expression of human recombinant ABHD11 protein in HEK293 cell.

## RT-PCR and cloning

Human Brain Poly A+ RNA (Clontech, reference 636101) was used to generate first strand cDNA with Superscript II reverse transcriptase (Invitrogen) and oligodT primers. Two μL of the cDNA synthesis reaction was prepared in a 50 μL final reaction volume for PCR amplification. The 50 μL reaction also contained 0.25 μΜ of each oligonucleotide primer and 2 IU of Taq polymerase Phusion® DNA Polymerase (Finzymes). The primer sequences were: 5' GCCGCCACCATGCGAGCCGGCCAACAG 3' (sense primer) – 5’ TTAGACCAGGAAGCCTCGG 3' for ABHD11 (reverse primer) and 5' GCCGCCACCATGGACTACAAAGACGATGACGACAAACGAGCCGGCCAACAGCTT 3' (reverse primer with C-terminal FLAG tag). Thirty-five cycles of PCR amplification were performed. Each cycle consisted of: denaturation 30 sec 98°C, annealing 30 sec 60°C and enzymatic extension 120 sec 72°C. After PCR, ABHD11 PCR-product DNA was electrophoresed on 2% agarose gels and visualized after ethidium bromide staining by UV fluorescence.

## Plasmid construction

All PCR products were purified from agarose gels using GFX micro plasmid prep kit (Amersham) and inserted into the pcDNA3.1A 5-His TOPO TA plasmid (Invitrogen) by TOPO cloning strategy for the direct insertion of Taq-polymerase- amplified PCR products into a plasmid vector 2.2.

## cDNA synthesis of the sequence encoding full length human ABHD11

To generate the cDNA a reverse transcriptase isolated from a MMLV retrovirus was used (Superscript III First-Strand Synthesis System for RT-PCR, Invitrogen). As with other polymerases, a short double-stranded sequence is needed at the 3' end of the mRNA to act as a start point for the polymerase. This is provided by the poly (A) tail found at the 3' end of most eukaryotic mRNAs to which a short complementary synthetic oligonucleotide (oligo dT primer) is hybridized (polyT-polyA hybrid). cDNA synthesis was performed according to the recommendation of the enzyme provider (Invitrogen) using 10 g of total human brain RNA (Clontech) primed with oligo(dT), together with all 4 deoxynucleotide triphosphates, magnesium ions and at neutral pH. The reverse transcriptase thus allows synthesizing a complementary DNA on the mRNA template. Each mRNA molecule in the mixture with a poly(A) tail can be a template and will produce a cDNA in the form of a single stranded molecule bound to the mRNA (cDNA:mRNA hybrid). The cDNA was then converted into a double stranded DNA. The first-strand cDNA obtained from the above synthesis reaction may be amplified directly by PCR. The enzyme was used according to the recommendations of the provider (Phusion) in HF buffer .

## Cloning of full length ABHD11 in mammalian cell expression vector

A PCR experiment was performed on the mixture of cDNA obtained as described in paragraph 3.1. The full length ABHD11 gene was specifically amplified with the primers GCCGCCACCATGCGAGCCGGCCAACAG and TTAGACCAGGAAGCCTCGG primers in a PCR reaction of 30 cycles in the following conditions: 1 X Phusion HF Buffer, 2 μL of cDNA template, 0.5 μΜ primers, 200 μΜ dNTPs and 1 unit of Phusion DNA Polymerase in a total reaction volume of 50 μL. Each cycle was composed of the following steps: Denaturation for 30 sec 98°C, hybridizing for 30 sec 60°C and synthesis for 2 min 72°C. The first step was preceded of an additional denaturation step of 2 minutes at 98°C. The DNA fragment obtained was cloned in the pCDNA3TOPO plasmid (Invitrogen).

# Expression of full length ABHD11 in mammalian cells

The expression experiments were carried out using the FreeStyle™ 293 expression system (Invitrogen). The FreeStyle™ 293 Expression System is designed to allow large-scale transfection of suspension 293 human embryonic kidney (HEK) cells in a defined serum-free medium. Transfection and culture protocols described in the user Manual of the kit were stringently observed. After the expression period, usually about 72 hours, the cells were washed with DPBS and pelleted. The samples were analyzed extemporaneously or frozen at -80°C immediately.

# Quantitative PCR for expression profile of ABHD11 and DAG lipase alpha and beta

RNA polyA and samples were obtained from Clontech (purified Premium Poly A+ RNA). Clontech Universal reference total RNAs were total RNA controls derived from whole tissue sources. First strand cDNA was generated by the superScript VILO cDNA synthesis Kit (1 µg RNA per reaction). The 5X VILO reaction mix contained random primers, oligodT, MgCl2, dNTPs and RNase out recombinant ribonuclease inhibitor. For each sequence a specific gene expression assay was designed using Applied Biosystems website for mRNA probe. Each gene expression assay contained a FAM-labeled TaqMan MGB probe and two PCR primers (250 nM probe and 900 nM each primer). PCR amplification were performed using 20 ng cDNA, TaqMan universal PCR master mix with AmpliTaq Gold enzyme (2X) and TaqMan Gene expression assay in 20 µL by sample. Standard run conditions: 2 min 50°C, 10 min 95°C and 40 cycles of 15 sec 95°C, 1 min 60°C. Quantitative analyses were performed using Applied Biosystems 7900HT Real time PCR system. RPLP0 gene was used as endogenous control

# Mouse embryonic fibroblasts preparation and mitochondrial respiration

14-days old embryos were harvested from C57Bl6-ABHD11 WT or KO transgenic mice. Head and liver were discarded before homogenization of the embryos by 10 drops of syringe in culture medium (DMEM + glutamax, 10% FBS, 1% Penicillin/Streptomycin) and incubated at 37°C, 5% CO2. The medium was changed every day, cells were splitted twice a week and according to genotyping, only homozygote cells were maintained. MEF cells were then seeded in a Seahorse collagen-coated 96w plate at 30 000 cells/well/80 µL in culture medium and allowed to adhere overnight. The medium was then replaced by 175 µL of SH assay medium (Agilent) + 1 mM Sodium pyruvate + 4.5 g/L glucose and pH adjusted to 7.4 at 37°C. Seahorse XFe96 was used to evaluate the mitochondrial respiration profile with 1.5 µM Oligomycine, 1 µM FCCP and 0.5 µM Rotenone/Antimycine.

# Mitochondria isolation from mouse liver

The liver was extracted from mice after cervical elongation and briefly rinsed in physiologic serum, cut in small pieces and transferred in 10 mL de MSHE+BSA (5 mM HEPES pH 7.2 (KOH), 70 mM Sucrose, 210 mM D-Mannitol, 1 mM EGTA, 0.5% BSA fatty acid free and pH adjusted at 7.2 at 37°C). Pieces were then slowly homogenized in a Dounce on ice and centrifuged 10 min at 800g, 4°C. The supernatant was rapidly transferred in a new tube and centrifuged 10 min at 8000g, 4°C. The pellet containing mitochondria was resuspended in 5 mL of MSHE+BSA and centrifuged 10 min at 8000g, 4°C. This final pellet was resuspended with a minimum volume of MSHE+BSA and kept for determination of protein concentration.

# Mitochondrial respiration in isolated mitochondria using XFe96 Seahorse

Mitochondria suspensions were diluted to have a final concentration of 4 µg per well in 25 µL, distributed in a Seahorse 96w plate, centrifuged 20 min at 2000g, 4°C and 155 µL of MAS+S+BSA per well was carefully added (2 mM HEPES pH 7.2, 70 mM Sucrose, 220 mM D-Mannitol, 10 mM KH2PO4, MgCl2 5 mM, 1 mM EGTA, pH 7.2 at 37°C). Just before use, 0.2% fatty acid free BSA, 10 mM Sodium Pyruvate and 5 mM Malate were added. The plate was allowed to equilibrate at 37°C without CO2 for 1h before launching the Seahorse assay with final concentrations of 4 mM ADP, 3 µM Oligomycine, 4 µM FCCP, 4 µM Antimycine/Rotenone.

# Preparation of mouse primary hepatocytes

The liver of anesthetized WT or KO ABHD11 transgenic mice was perfused through the inferior vena cava with prewarmed Liver Perfusion medium (Gibco) followed by prewarmed Liver Digest medium (Gibco). Liver was then transferred on a culture plate with 15 mL of cold L-15 medium, torn with tweezers in sterile conditions and gently shaken to release hepatocytes. Cell suspension was filtered through a 100 µm cell strainer and pelleted for 5 min at 50g, 4°C. After 2 rounds of washes in Hepatocyte Wash medium (Gibco) and centrifugations, primary hepatocytes were resuspended in William’s medium plus 5% FBS and 1% Pen/Strep and counted to estimate cell viability. Use preparations only when viability >50%.

# Mito stress test of primary hepatocytes with Seahorse (Agilent)

Mouse primary hepatocytes were seeded at 10 000 cells/well /100µL in a Seahorse collagen-coated 96w plate in William’s medium plus 5% FBS and 1% Pen/Strep and incubated overnight at 37°C. Culture medium was then gently replaced by 175 µL/well of assay medium: XF base medium (Agilent), 1 mM sodium pyruvate, 1 mM glutamine, 2 g/L glucose and pH adjusted to 7.4 at 37°C. The plate was incubated for 1h at 37°C without CO2 and loaded in Seahorse XFe96 with a cartridge containing mitostress compounds to final concentrations of 2 µM Oligomycine, 1 µM FCCP and 0.5 µM Rotenone/AntimycineA.

# Beta oxidation of primary hepatocytes with XFe 96 Seahorse

Mouse primary hepatocytes were seeded in a Seahorse collagen-coated 96w plate at 8000c/well in William’s medium plus 5% FBS and 1% Pen/Strep. After 4h, the medium was changed for fresh substrate limited medium: XF base medium (Agilent), 0.5 mM Glucose, 1 mM Glutamine, 0.5 mM L-carnitine, 0.5% FBS and pH adjusted to 7.4 at 37°C. Cells were gently rinsed 2 times with this medium and incubated for 20 hours at 37°C without CO2. The medium was then changed for FAO medium: KHB plus 2.5 mM Glucose, 1 mM Glutamine, 0.5 mM L-Carnitine, 5 mM HEPES pH 7.4 at 37°C, where KHB buffer contains: 111 mM NaCl, 4.7 mM KCl, 1.25 mM CaCl2, 2 mM MgSO4, 1.2 mM NaH2PO4. Cells were rinsed two times in FAO medium and incubated 15 min at 37°C without CO2. Half of the plate was treated by Etomoxir (40µM final concentration) and incubated for 45mn at 37°C without CO2. Add 30 µL of palmitate-BSA or BSA control (Agilent) in cell plate just before launching the run in Seahorse XFe96. The cartridge was filled with mitostress reagents to have final concentration of 2 µM Oligomycine, 2.5 µM FCCP and 2 µM Rotenone and 4 µM Antimycine, all diluted in FAO buffer.

# Biochemical mice blood analysis

The following parameters were analyzed on Cobas6000® analyzer (Roche) : HDL, LDLC, AST, ALT, GGT, albumin, glucose, cholesterol, triglyceride, urea, creatinine, ALP, Phosphor, Calcium, total protein, total bilirubin.

# Mice behaviors phenotyping

Behavior mice phenotyping, called SHIRPA (SmithKline Beecham Pharmaceuticals, Harwell, MRC Mouse Genome Centre and Mammalian Genetics Unit, Imperial College School of Medicine at St Mary's, Royal London Hospital, St Bartholomew's and the Royal London School of Medicine, Phenotype, Assessment) has been performed on WT and KO ABHD11 mice at 9th week of age. The SHIRPA allows the detection of anomalies that could affect mice behaviors in 35 different tests including body position, locomotion, salivation, lacrimation, tremor detection, vision and olfaction testing. This phenotyping includes biochemical analyses, locomotor test, muscular strength evaluation, sensory vision test and touch escape test.

# 2AG dosage in mice tissues and mice isolated mitochondria

## Samples preparation

Tissue samples (brain structures, liver, heart, muscle and pancreas) from individual mice were homogenized in a CK28 Precellys tube with 1 ml of a solution of ethyl acetate/n-heptane (1:1), containing 50 ng of 2-arachidonoyl glycerol-d8. The samples were grinded three times at 4000 rpm for 90 sec. The homogenate was centrifuged at 4°C (4500 g for 5 min) and 950 µL of the supernatant were transferred to a borosilicate tube. The sample was grinded again in 1 mL of a solution of ethyl acetate/n-heptane (1:1) three times at 4000 rpm for 90 sec. After centrifugation, 950 µL of the supernatant were transferred to the same borosilicate tube. The combined extract was evaporated to dryness at 37°C under a stream of nitrogen. The dried residue was reconstituted in 150 µL of methanol with 5mM ammonium acetate and 150 µL of water with 5 mM ammonium acetate, of which 100 µL was used for analysis by online solid phase extraction coupled with liquid chromatography and tandem mass spectrometry (XLC-MS/MS).

Isolated mitochondria from liver of individual mice were extracted using 250 µL isolated mitochondria with 1 ml of a solution of ethyl acetate/n-heptane (1:1), containing 50 ng of 2-arachidonoyl glycerol-d8. Samples were vortexed centrifuged (2300g for 5 min) and the supernatant was transferred to a borosilicate tube. After evaporation to dryness at 37°C under a stream of nitrogen, the dried residue was reconstituted in 125 µL of methanol with 5mM ammonium acetate and 125 µL of water with 5 mM ammonium acetate, of which 200 µL was used for analysis by online solid phase extraction coupled with liquid chromatography and tandem mass spectrometry (XLC-MS/MS).

Adipose tissue samples (50-100 mg) from individual mice were homogenized in a CK28 Precellys tube with 0.3 ml of a solution of ethyl acetate/n-heptane (1:1), containing 50 ng of 2-arachidonoyl glycerol-d8. The samples were grinded three times at 4000 rpm for 90 sec. The homogenate was then loaded on a silica column, previously conditioned with 2 x 5 mL of n-heptane. After two successive washes, with 2 x 5 mL of n-heptane and 5 mL of dichloromethane/n-heptane (1:1), the column was eluted with 5 mL of dichloromethane/methanol (1:1) and 5 mL of methanol. Both eluates were combined in a borosilicate tube and evaporated to dryness at 37°C under a stream of nitrogen. The dried residue was reconstituted in 150 µL of methanol with 5mM ammonium acetate, of which 125 µL were combined with 125 µL of water with 5 mM ammonium acetate. 200 µL was used for analysis by online solid phase extraction coupled with liquid chromatography and tandem mass spectrometry (XLC-MS/MS).

## Analytical Method

Samples were injected into a SPE cartridge (2 mm inside diameter · 1 cm length, packed with C18-HD stationary phase), part of a SPE platform from Spark Holland (Emmen, The Netherlands). The samples were then cleaned and preconcentrated with successive methanol⁄water washes. Thereafter the SPE cartridge was directly eluted on an Symmetry C18 HPLC column (2.1 x 50 mm; 3.5 µm) from Waters (Milford, MA, USA) with a water/methanol with 5 mM ammonium acetate gradient (60% B for 2 min, 60 to 95 %B in 3 min, 95% B for 7 min and back to initial conditions), at a flow rate of 0.2 mL ⁄min to the mass spectrometer.

2-AG and internal standard (d8-2-AG) were analyzed on a Quantum Ultra triple quadrupole (Thermo Electron Corporation, San Jose, CA, USA). Positive electrospray was performed on a Thermo IonMax ESI probe. To increase the sensitivity and specificity of the analysis, we worked in multiple reaction monitoring and followed the MS⁄MS transitions: 2-AG MNH4+, 396.2–287.2; d8-2-AG MNH4+, 404.2–294.2.

The spray chamber settings were as follows: heated capillary, 350°C; spray voltage, 4000 V; sheath gas, 60 arbitrary units; auxiliary gas 20 arbitrary units. Calibration curves were produced by using synthetic 2-AG (Cayman Chemical, Ann Arbor,MI). The amounts of 2-AG in the samples were determined by using inverse linear regression of standard curves. Values are expressed as ng per mg of tissue or ng/mg of proteins for mitochondria.

# RNA extractions and qRT-PCR analysis from mice tissues

After dissection and rinsing with saline solution, tissues were immersed and stored in 1 mL of RNA later at -80 ° C. On the day of extraction, the tissues were dried and immersed in 750 μL Qiazol lysis reagent. Homogenization with CK14 tubes (Precellys) was performed with 2 X 10 sec at 5000 rpm with 20 sec break between cycles. 140 μl chloroform were added and vortexed during 15 sec and centrifuged at 12000 g for 15 minutes at 4 °C. The aqueous phase (app 350μl) was recovered and performed using a custom protocol on Qiaextractor robot (Qiagen) with the Rneasy 96 kit (Qiagen) plus one step of DNAse treatment and a final elution in 70 μL H2O RNase free. Total RNA quality was analyzed on an RNA LabChip (Agilent Technologies) using a 2100 Bioanalyzer and quantified using an Xpose spectrophotometer (Trinean NV, Belgium).

Two μg of total RNA were reverse transcribed by High Capacity cDNA Archive Kit (Applied Biosystems) and oligo(dT) 16 (Sigma-Aldrich) at 2.5 µM final concentration. Samples were incubated for 10 min at 25°C followed by 120 min at 37°C then heated at 95° C. For QPCR experiment, Fast SYBR Green Master Mix or TaqMan Universal Mix (Thermo Fisher Scientific) and ABHD11 Taqman probe Mm00460922_m1 (Thermo Fisher Scientific) or FABP6 QuantiTect Primer Assay QT00133000 (Qiagen) or FGF15 home design (Sigma-Aldrich) were amplified and normalized with housekeeping gene Rpl37a QT00252266 (Qiagen). Real-time PCR instrument was an ABI Prism 7900 sequence detector (Applied Biosystems) with SDS 2.4.1 software for analysis. For qPCR expression, cycle threshold (Ct) values of each sample were converted to Delta Ct: $\Delta\text{Ct}\text{ }\text{(studied gene)}$ = ((Ct studied gene–Ct Rpl37a (Housekeeping gene)). For histogram graphs, results are presented in – (delta ct) or arbitrary units *100 with AU*100 = $\text{100 * 2e}^{-\text{∆Ct(studied gene)}}$. Statistical analysis was done in one way anova Newman Keuls for factor group on AU.

# Western blot

For mitochondrial enriched fractions from mouse embryonic fibroblasts : MEFs were prepared from WT and KO embryos as previously described and Qiagen QProteome mitochondria isolation kit was used according to the manufacturer. All fractions were analyzed for ABHD11 first by electrochemiluminescence (ECL) and membranes were then re-exposed to COX IV-HRP antibody and beta-Tubulin-HRP antibody from Cell Signaling.

For primary hepatocytes and for liver isolated mitochondria: Lysates were prepared in PIERCE IP lysis buffer completed by anti-proteases/phosphatases and centrifuged at 14000g for 10 min at 4°C. Lysates were loaded (20 µg) on SDS-PAGE, transferred on nitrocellulose membranes and submitted for Western blot for ABHD11, beta-Tubulin and COX IV. ABHD11 antibody from Santa Cruz, 138080, beta-Tubulin-HRP antibody and COX IV-HRP antibody from Cell Signaling.

For ileum or liver of DIO mice: The organs of each mouse (8 mice in each group) were extracted, rapidly frozen on dry ice and pieces were stored at -80°C. Each piece was transferred in Precellys CK28 tubes (Bertin Technologies) with PIERCE IP lysis buffer completed by anti-proteases/phosphatases and homogenized in Precellys 24 for 2X10s. The tubes were then centrifuged at 14000g for 10 min at 4°C. Supernatants were loaded (20 µg) on SDS-PAGE, transferred on nitrocellulose membranes and submitted for Western blot. FABP6 and alpha Tubulin were revealed by infrared and ABHD11 was revealed by ECL. Anti FABP6 polyclonal antibody was from Abcam (ab91184), mouse alpha Tubulin antibody by Cell Signaling (3873), ABHD11 antibody by Santa Cruz (138080), Tom20 antibody by Cell Signaling (13929). Secondary IRDyes antibodies were from Li-Cor and secondary HRP conjugated antibodies from GE Healthcare. Membranes were revealed using Odyssey-FC (Li-Cor) and proteins visualized and quantified using Image Studio (Li-Cor). For electro-chemiluminescence, membranes were exposed in LAS 3000, Fuji-film.

# Composition of STD and HFD foods:

STD (=control) and HFD (=TD.97366)
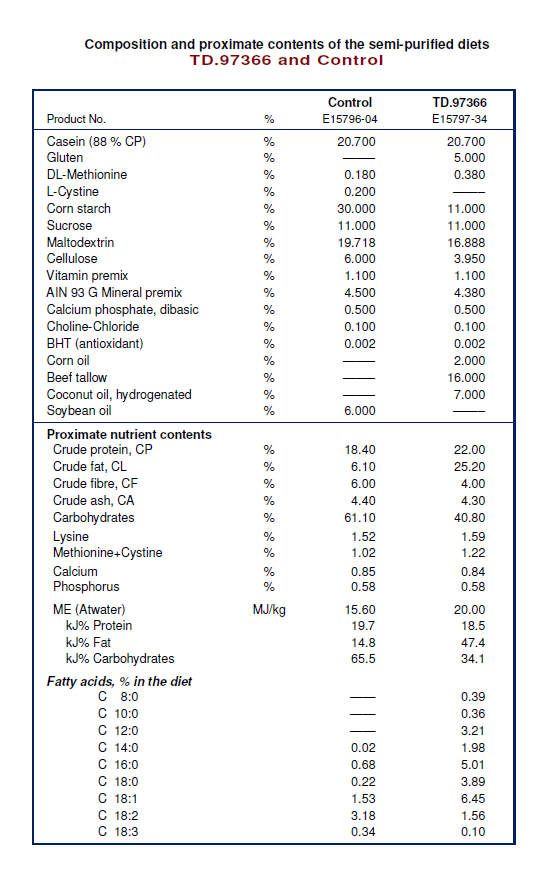


1. Jessani, N. and B.F. Cravatt, *The development and application of methods for activity-based protein profiling.* Curr Opin Chem Biol, 2004. **8**(1): p. 54-9.
