# Supplementary material for: ABHD11, a new diacylglycerol lipase involved in weight gain regulation
Source: PLoS One. 2020 Jun 24;15(6):e0234780. doi: 10.1371/journal.pone.0234780 (PMC7313976; doi:10.1371/journal.pone.0234780)
Supplement: S3 Appendix — (PDF) [file pone.0234780.s003.pdf]

Figure 2

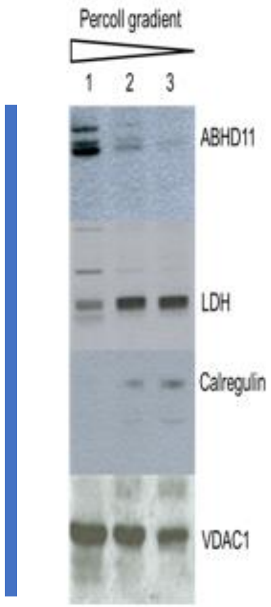

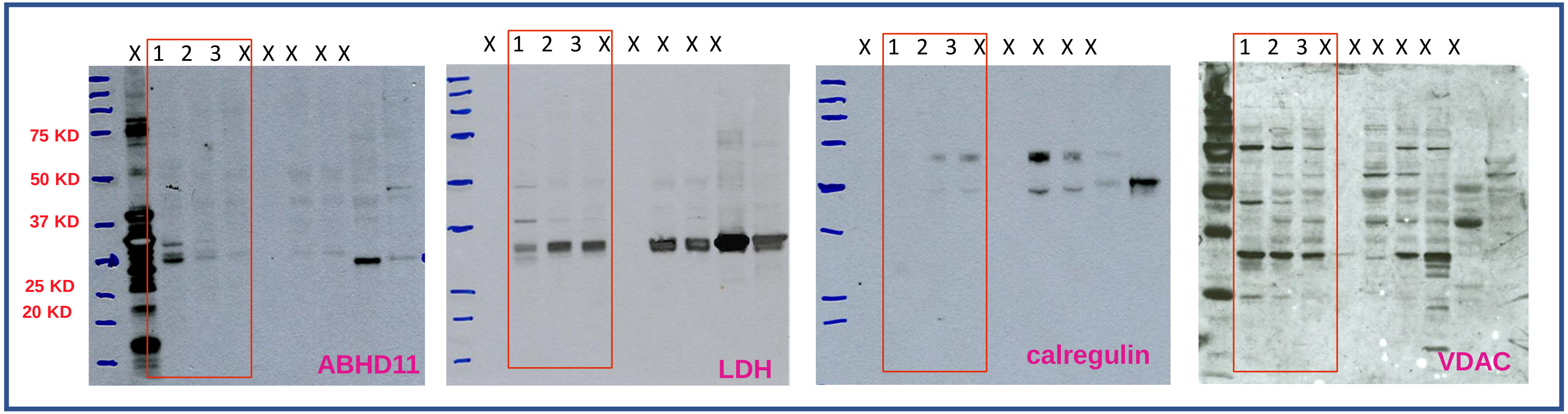

Raw image for Figure 2 : WB. Blot scan performed on a scanner Typhoon (Amersham)

figure S1-B blot A

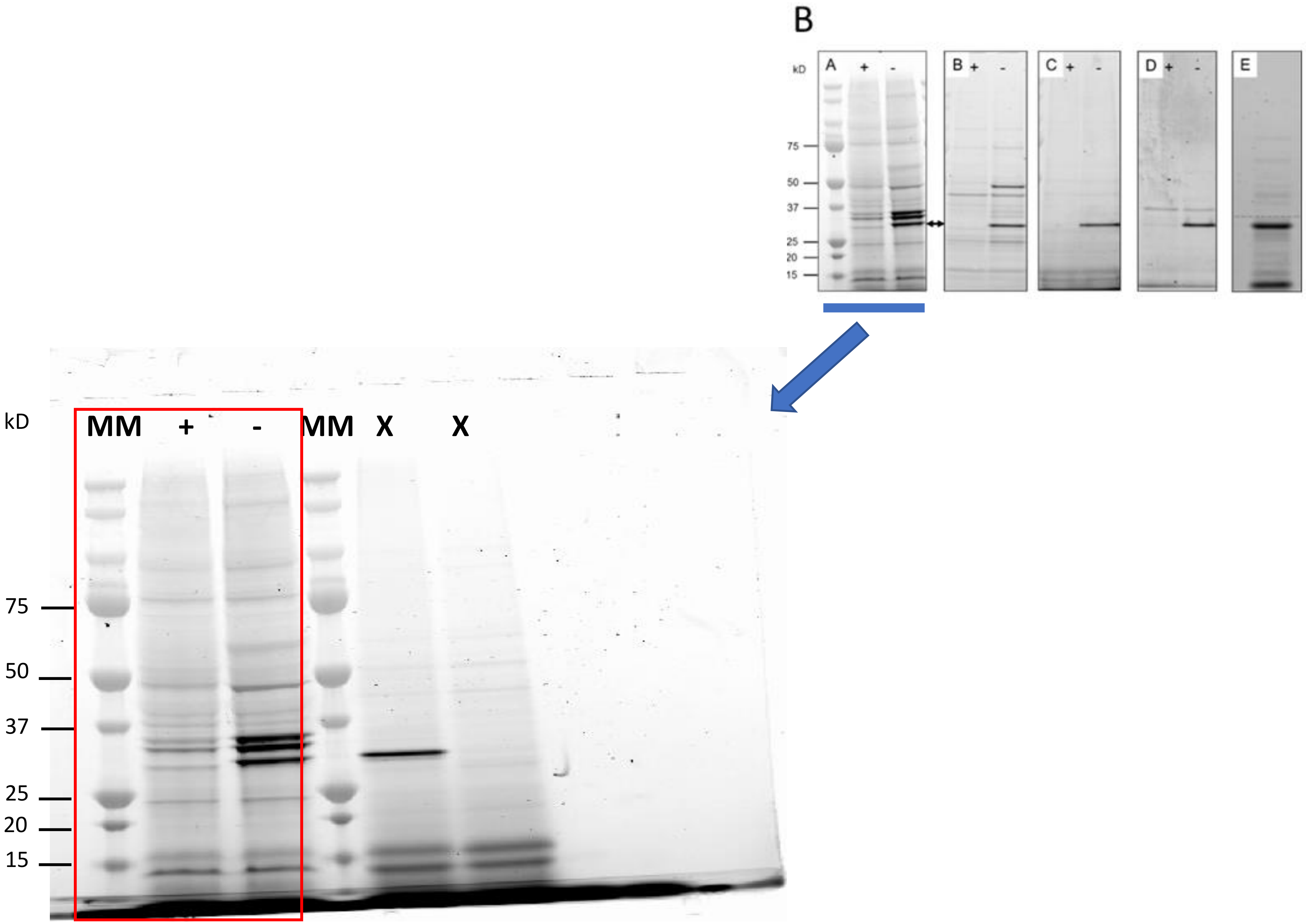

Raw blot figure S1-B blot A. Blot scan performed on a scanner Typhoon (Amersham)

Figure S1-B blot B

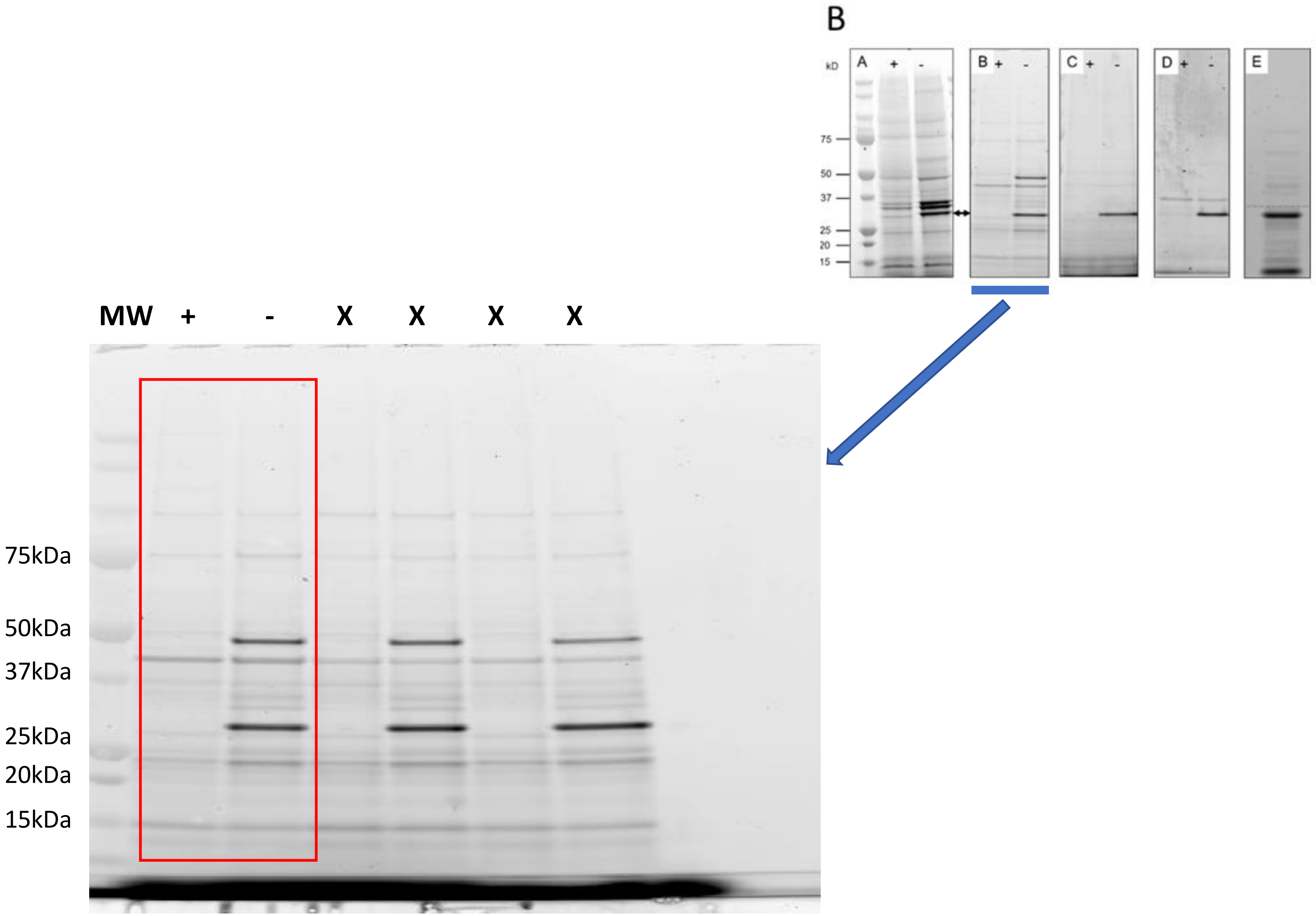

Raw blot figure S1-B blot B. Blot scan performed on a scanner Typhoon (Amersham)

figure S1-B blot C

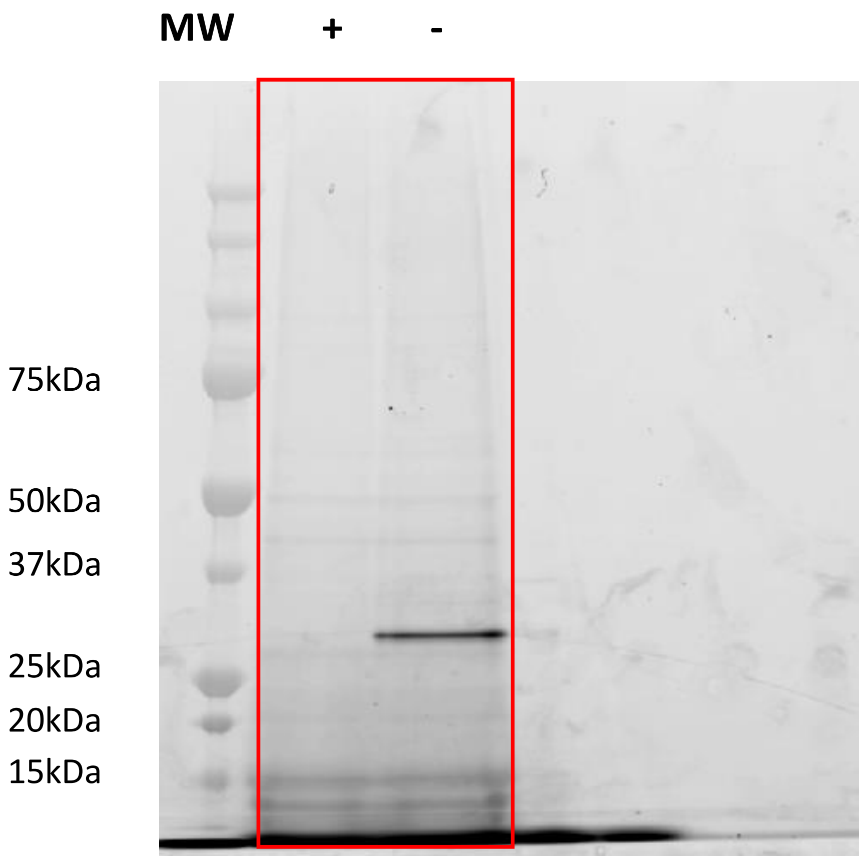

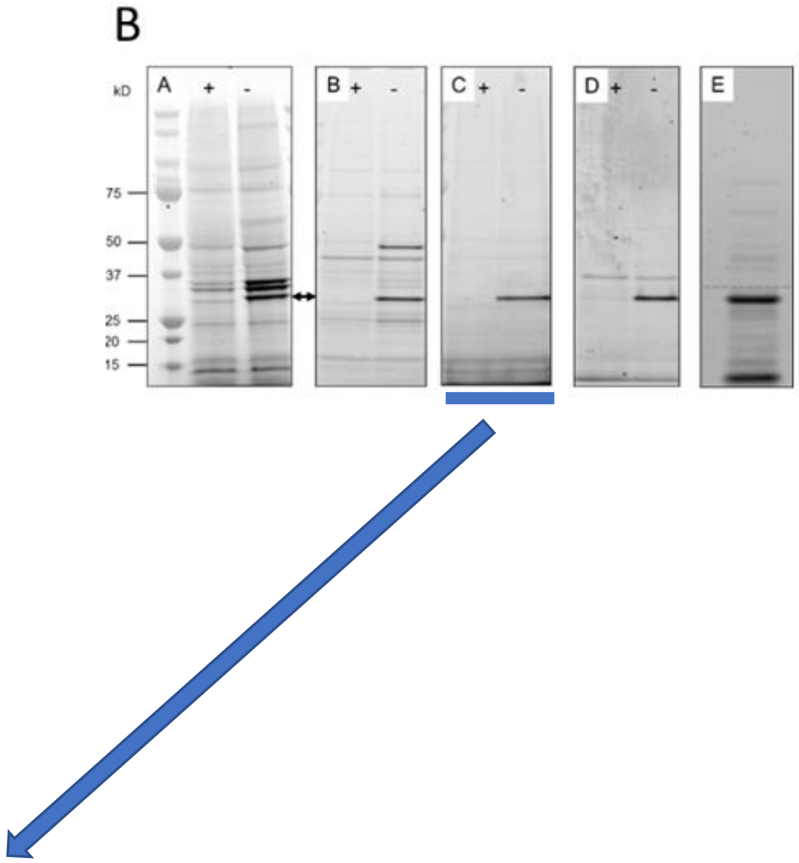

Raw blot figure S1-B blot C. Blot scan performed on a scanner Typhoon (Amersham)

figure S1-B blot D

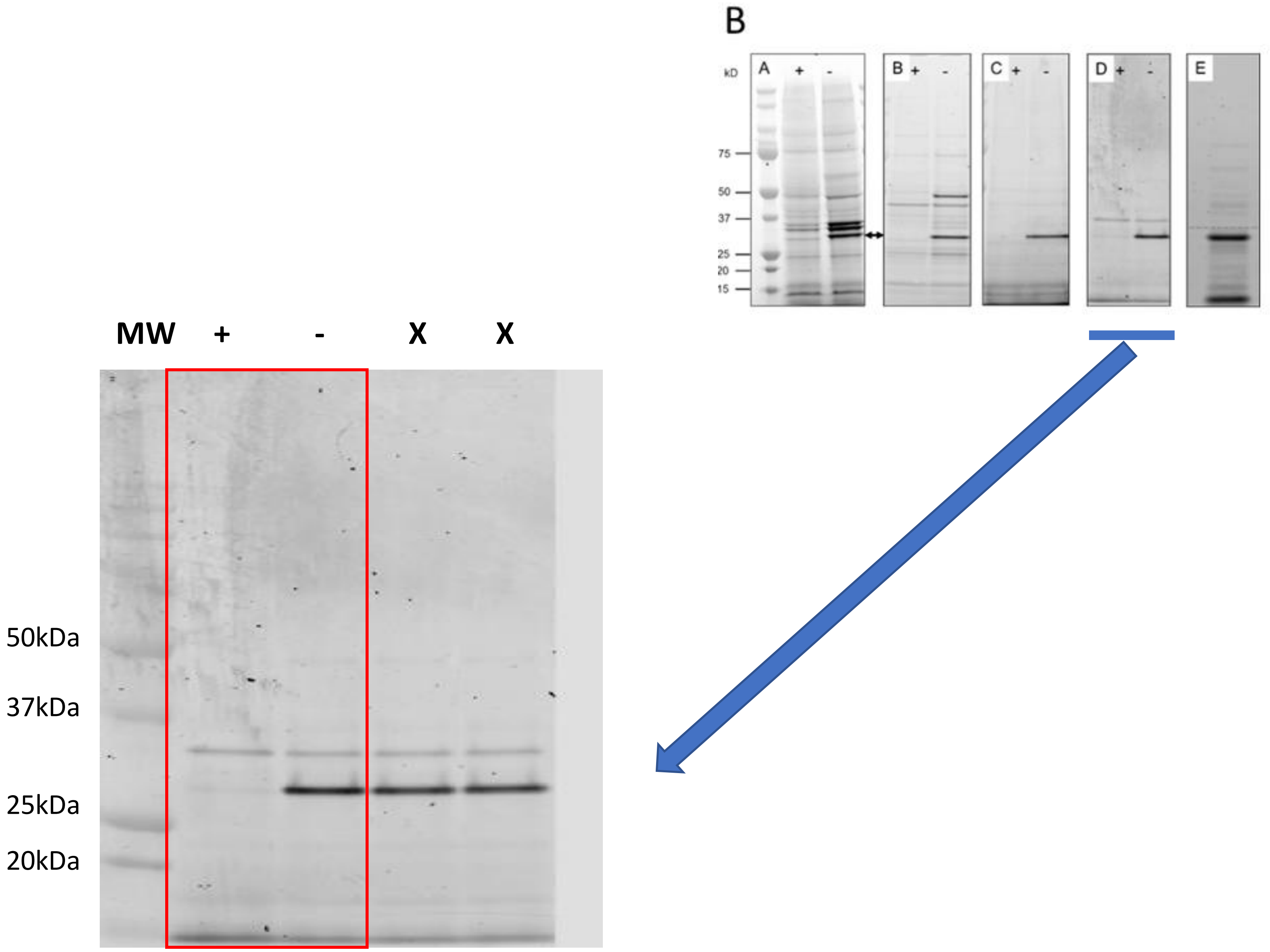

Raw blot figure S1-B blot D. Blot scan performed on a scanner Typhoon (Amersham)

figure S1-B blot E

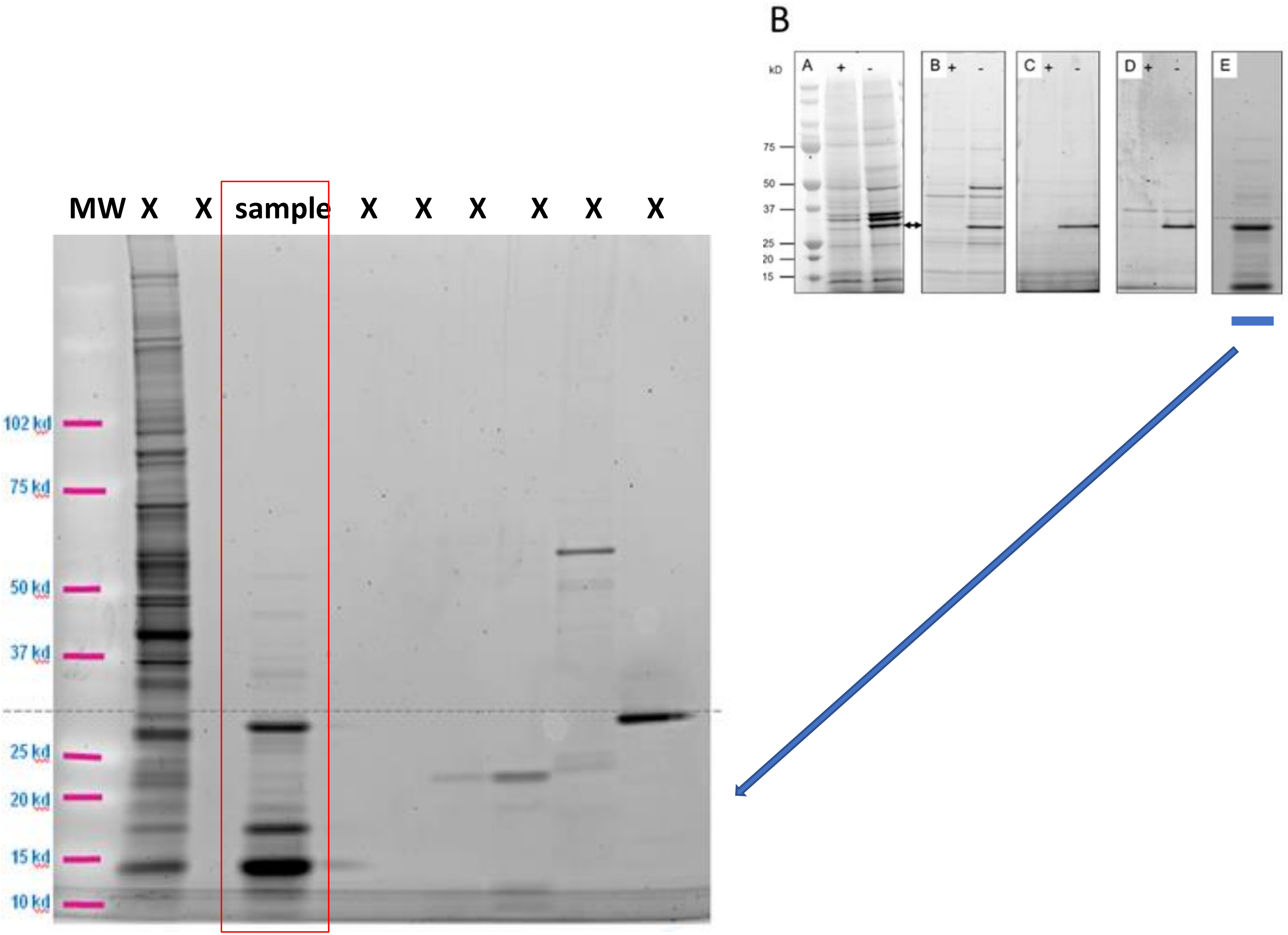

Raw blot figure S1-B blot E. Blot scan performed on a scanner Typhoon (Amersham)

figure S1-C

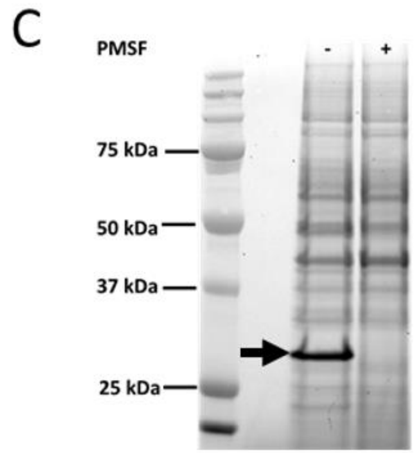

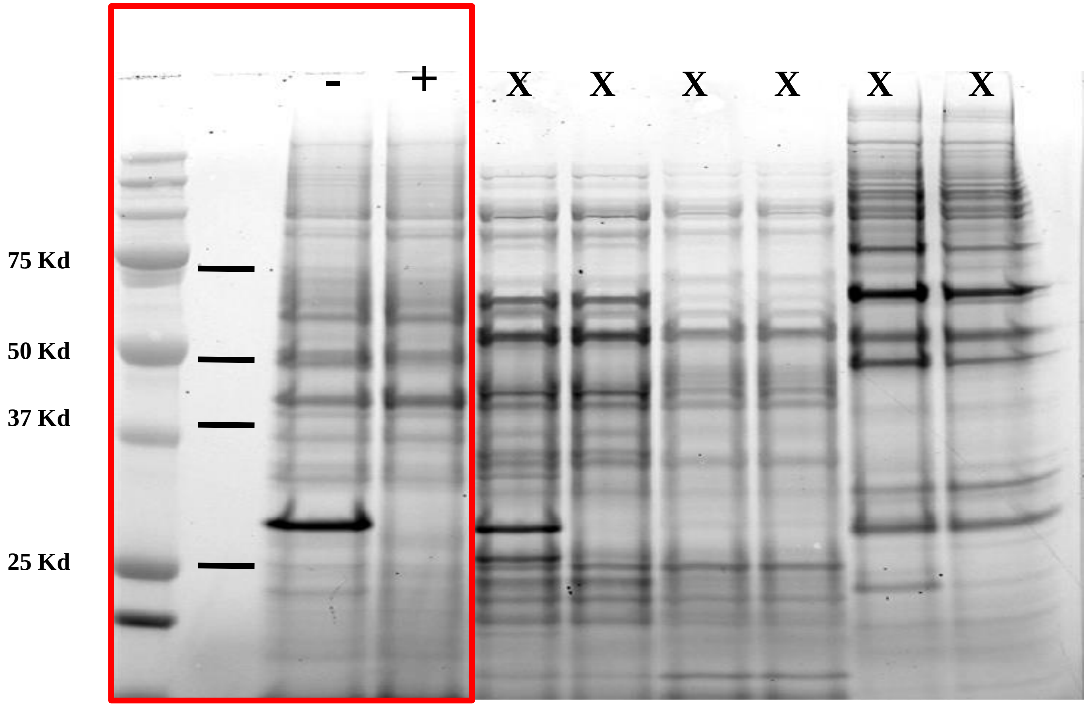

Raw blot figure S1-C. Blot scan performed on a scanner Typhoon (Amersham)

Figure S3C

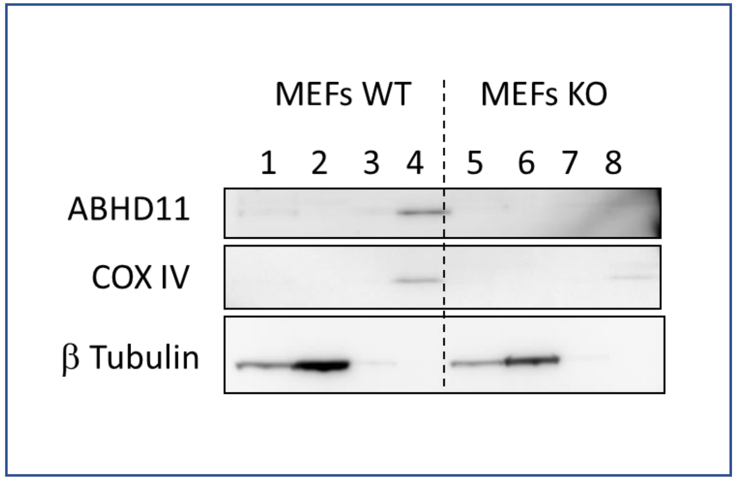

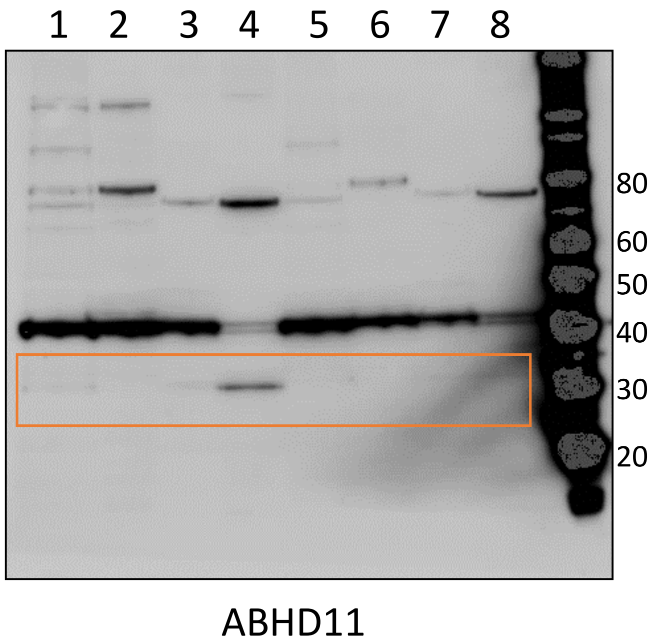

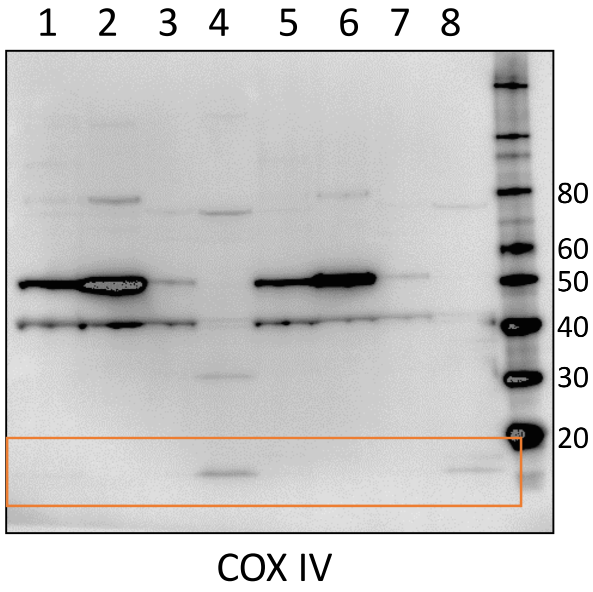

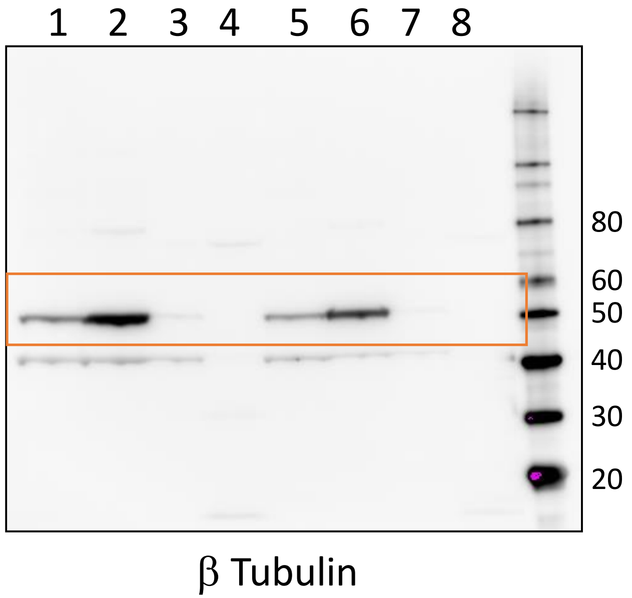

Raw images figure S3-C: Blot scan performed with imager LAS3000, FujiFilm. ECL

Figure S4B

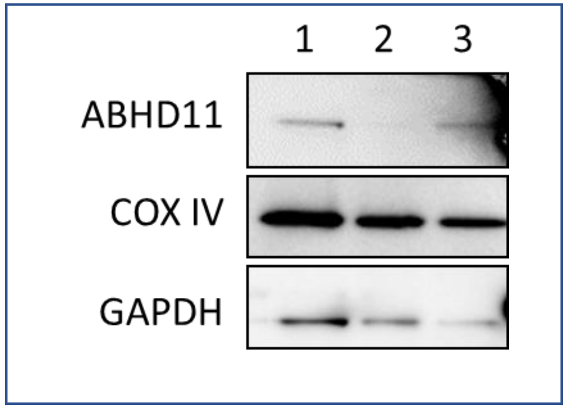

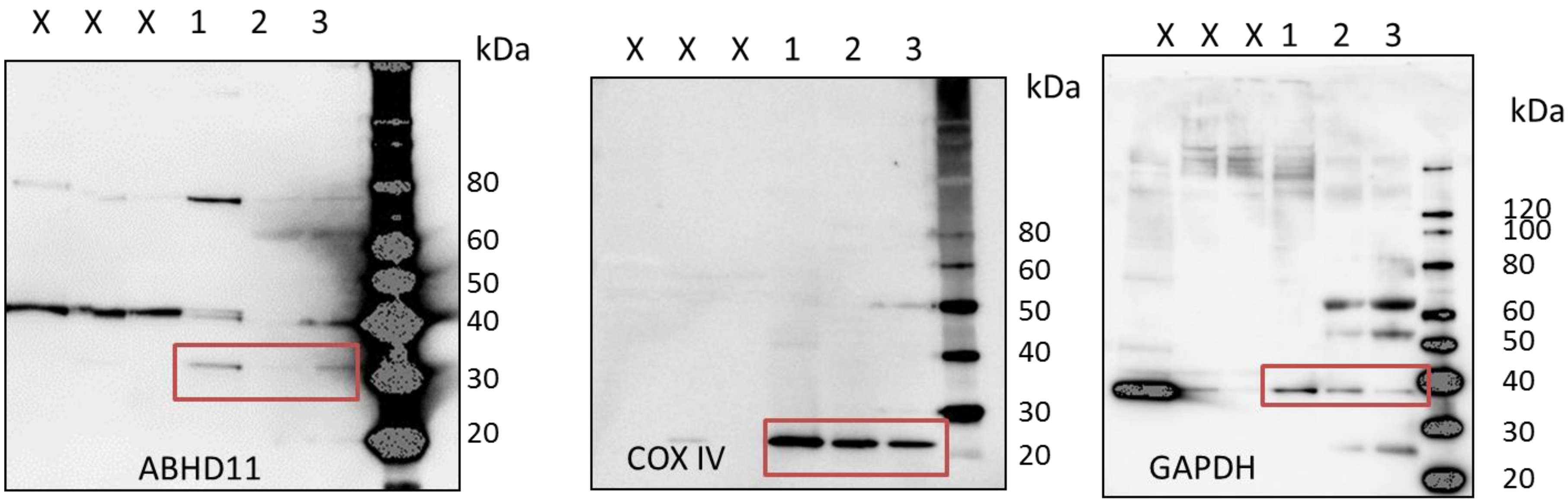

Raw images figure S4-B: Blot scan performed with imager LAS3000, FujiFilm. ECL

Figure S5E

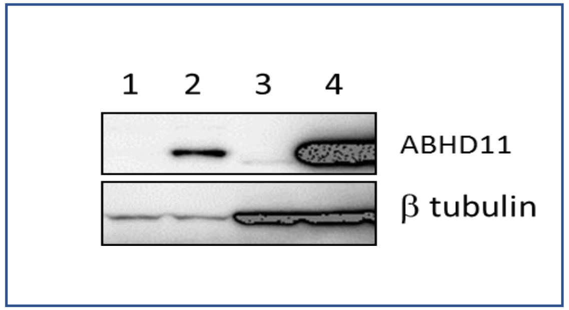

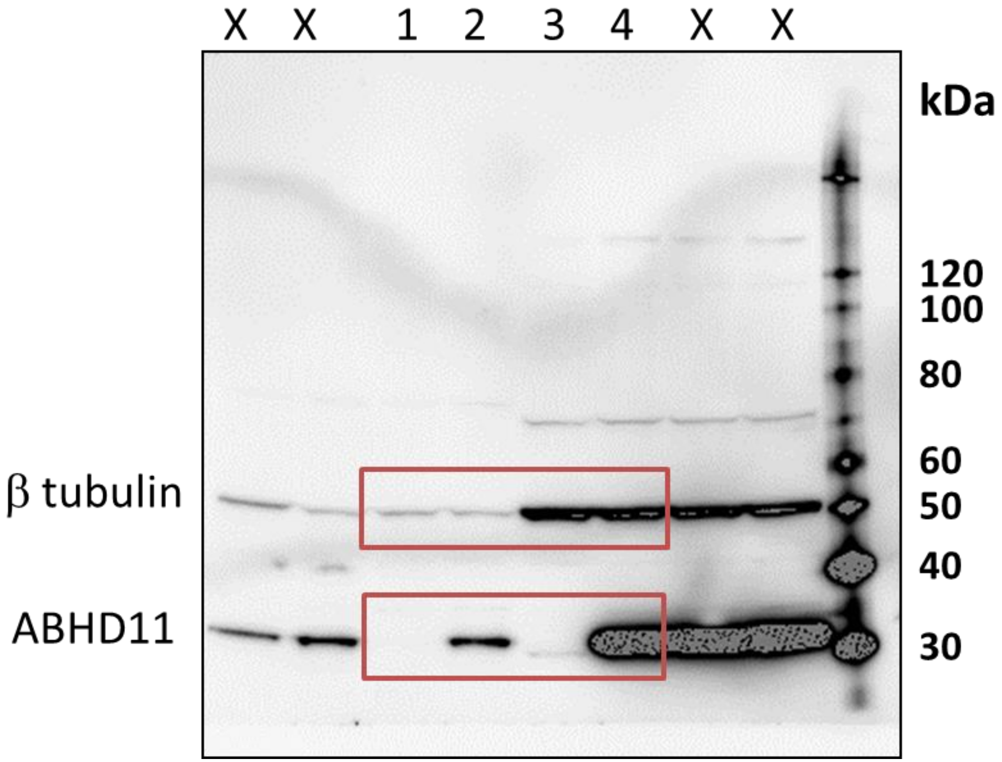

Raw image figure S5-E: Blot scan performed with imager LAS3000, FujiFilm. ECL

Figure S13 A: ileum lysates

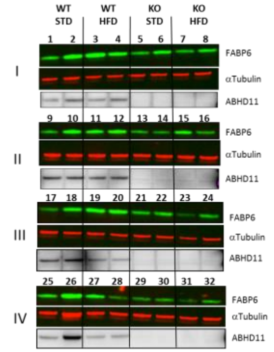

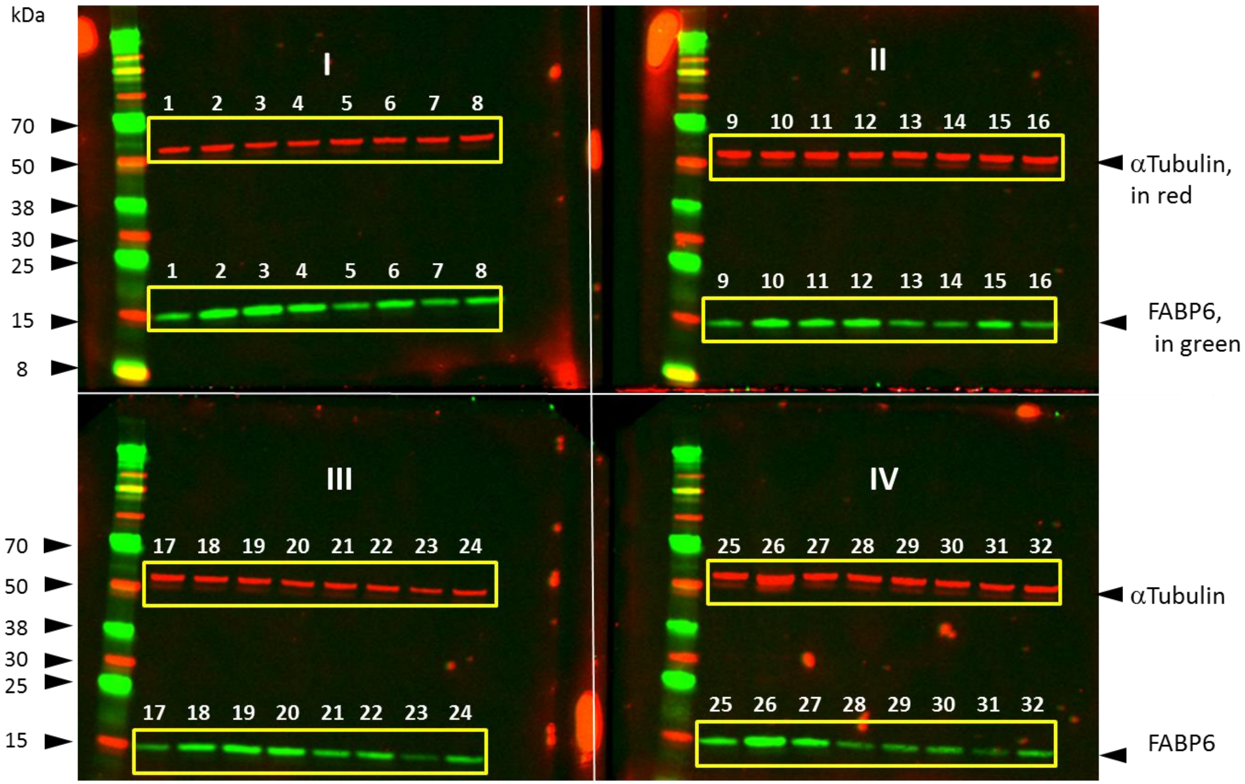

Raw images of the 4 membranes I, II, III and IV in fluorescence (IR) for aTubulin (Red) and FABP6 (green) protein expression. Marker: Chameleon Duo Pre-stained protein ladder (Li-Cor). Blot scan performed with Odyssey-FC, Li-Cor

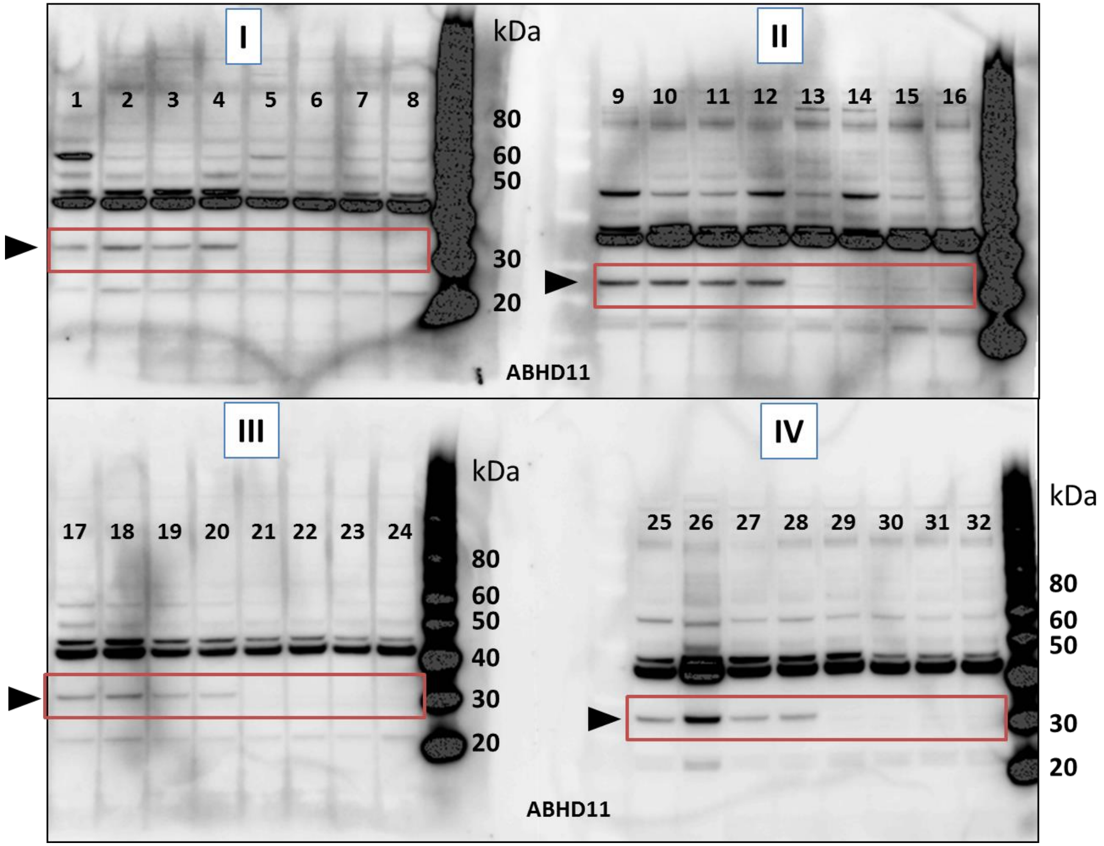

Raw images of the 4 membranes I, II, III and IV for ABHD11 protein expression. Marker Magic mark XP protein standard. Blot scan performed with LAS 3000, FujiFilm. ECL

Figure S14C: liver lysates

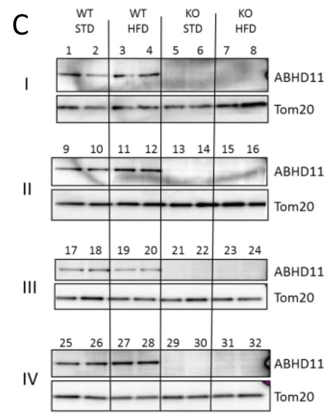

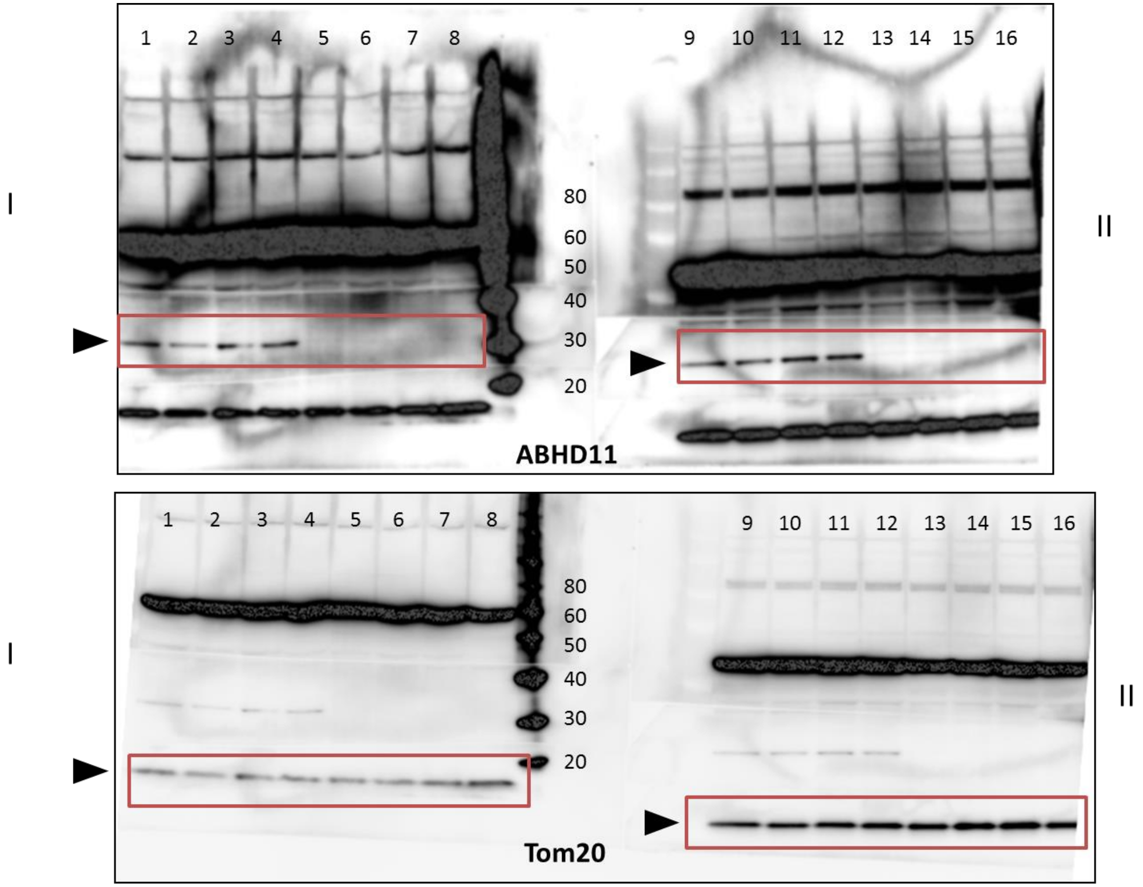

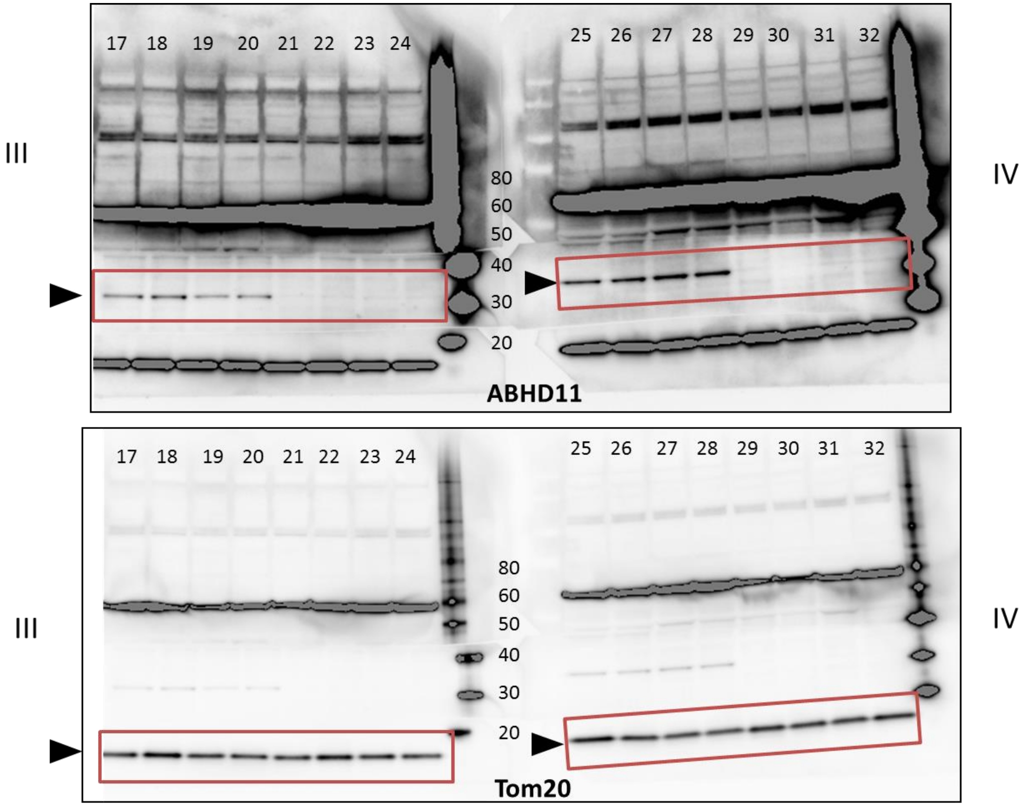

Raw images of the 4 membranes I, II, III and IV for ABHD11 and Tom20 protein expression. Marker Magic mark XP protein standard. Blot scan performed with LAS 3000, FujiFilm. ECL
